# DFF-ChIP: a method to detect and quantify complex interactions between RNA polymerase II, transcription factors, and chromatin

**DOI:** 10.1093/nar/gkae760

**Published:** 2024-09-09

**Authors:** Benjamin M Spector, Juan F Santana, Miles A Pufall, David H Price

**Affiliations:** Department of Biochemistry and Molecular Biology, The University of Iowa, Iowa City, IA 52242, USA; Department of Biochemistry and Molecular Biology, The University of Iowa, Iowa City, IA 52242, USA; Department of Biochemistry and Molecular Biology, The University of Iowa, Iowa City, IA 52242, USA; Department of Biochemistry and Molecular Biology, The University of Iowa, Iowa City, IA 52242, USA

## Abstract

Recently, we introduced a chromatin immunoprecipitation (ChIP) technique utilizing the human DNA Fragmentation Factor (DFF) to digest the DNA prior to immunoprecipitation (DFF-ChIP) that provides the precise location of transcription complexes and their interactions with neighboring nucleosomes. Here we expand the technique to new targets and provide useful information concerning purification of DFF, digestion conditions, and the impact of crosslinking. DFF-ChIP analysis was performed individually for subunits of Mediator, DSIF, and NELF that that do not interact with DNA directly, but rather interact with RNA polymerase II (Pol II). We found that Mediator was associated almost exclusively with preinitiation complexes (PICs). DSIF and NELF were associated with engaged Pol II and, in addition, potential intermediates between PICs and early initiation complexes. DFF-ChIP was then used to analyze the occupancy of a tight binding transcription factor, CTCF, and a much weaker binding factor, glucocorticoid receptor (GR), with and without crosslinking. These results were compared to those from standard ChIP-Seq that employs sonication and to CUT&RUN which utilizes MNase to fragment the genomic DNA. Our findings indicate that DFF-ChIP reveals details of occupancy that are not available using other methods including information revealing pertinent protein:protein interactions.

## Graphical Abstract

**Graphical Abstract:**
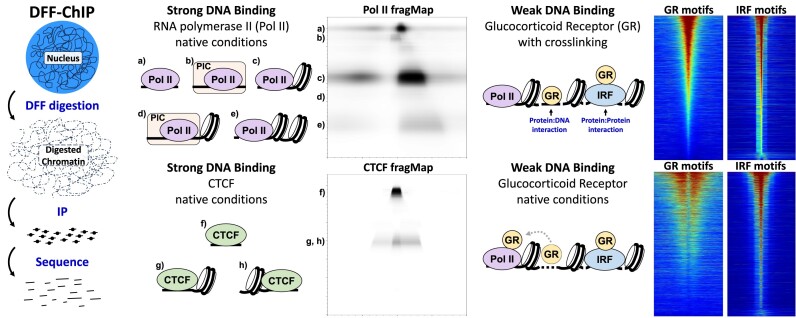

## Introduction

Regulation of gene expression is achieved through the complex interplay among transcription factors, the general transcription machinery, and the chromatinized genomic template. In this highly coordinated process, Pol II is directed to sites of initiation by the actions of general initiation factors to form preinitiation complexes over transcription start sites (TSSs). Transcription initiation by Pol II leads to the exchange of initiation factors for the pausing factors, DRB sensitivity inducing factor (DSIF) and the negative elongation factor (NELF). Together with the precisely positioned +1 nucleosome, this leads to the accumulation of promoter proximal paused complexes approximately 40 bp downstream of the TSS (1–3). The majority of these paused complexes terminate, but a fraction is released into productive elongation via the phosphorylation of DSIF by P-TEFb, the release of NELF, and the incorporation of the PAF1 complex and RTF1 (2–6). For transcription to be regulated in response to cellular conditions or environmental stimuli, sequence-specific transcription factors work to direct the recruitment and function of Pol II to specific promoters and enhancers by direct interactions with the Mediator complex (7,8). The 1.4 MDa Mediator complex is thought to function as a junction between Pol II PICs and transcription factors that often lie distal to the affected Pol II complex (9). Since this distance can be very short or very large, determining the exact function of specific transcription factor binding sites has been extremely difficult. Furthermore, the intrinsic affinity of transcription factors for specific DNA sequences is a poor predictor of occupancy. These issues emphasize that transcription factor function is highly context dependent. Indeed, it has been consistently shown that it is the combinatorial effect of multiple transcription factors coupled with the current state of chromatin that influence where and how transcription factors function (7,10). Therefore, methods that preserve protein:protein interactions are essential in further understanding how transcription factors function to regulate transcription.

The assessment of transcription factor occupancy has largely relied on chromatin immunoprecipitation followed by sequencing (ChIP-Seq). In the ChIP-Seq method, cells are crosslinked with formaldehyde, sonicated, and the soluble chromatin is incubated with antibodies to enrich DNA fragments bound by the target protein which are then sequenced. However, this method has limitations that have hindered a full determination of the sites of occupancy and how the DNA-bound factor interacts with neighboring proteins. Firstly, fragment sizes resulting from sonication range widely from ∼100–300 bp under the most extreme conditions to ∼200–600 bp with more standard sonication conditions (11,12). Since the binding footprint of a typical DNA binding protein falls far below these ranges, the sites of occupancy are not accurately determined. Furthermore, the harsh fragmentation conditions may disrupt protein:protein interactions that are important for dissecting the function of the targeted factor and lead to the potential false correlations relying on position rather than actual co-occupancy of factors and chromatin.

Advancements in the ChIP methodology have led to the usage of micrococcal nuclease (MNase) instead of sonication to fragment DNA (13). MNase nicks mainly unbound DNA and creates fragments when nicks are close to each other. This method has improved the resolution possible in ChIP methods and in a few cases has also enabled the assessment of occupancy without crosslinking under ‘native’ conditions (14,15). However, MNase has a preference for AT rich sequences and can cleave within DNA sites that are bound by transcription factors and nucleosomes (16–18). Additionally, MNase digests RNA, single stranded DNA, and has both endo- and exo-nuclease activity. Regardless of these issues, further advancement of MNase-ChIP has led to the development of the CUT&RUN method that allows for targeted MNase digestion and solublization only of regions near the target protein (19,20). In the CUT&RUN protocol, immobilized and permeabilized nuclei are incubated with the primary antibody of interest followed by incubation with a Protein-A-MNase fusion protein resulting in selective digestion of target regions following the introduction of calcium ions. This selective digestion and recovery of the genome substantially reduces background signal from across the genome. However, fragments recovered from this method are not subjected to immunoprecipitation enrichment, leaving the door open for recovery of nearby nucleosomes that may or may not be interacting with the target protein. Without an immunoprecipitation step, CUT&RUN cannot distinguish genuine chromatin interactions from unrelated nearby nucleosomes.

Here, we more fully describe a method, DFF-ChIP, that precisely detects chromatin complexes utilizing DFF, an endonuclease responsible for destruction of genomic DNA during apoptosis (21,22). DFF has excellent potential, compared to MNase, because it generates double strand breaks, does not significantly digest DNA within the footprint of bound proteins, lacks all exonuclease activity, and does not digest RNA (21–23). Recent work by our lab has established that DFF preserves contacts between general transcription factors and chromatin (1,18,24–26). To broaden the scope of DFF-ChIP, and determine its efficacy of recovering protein:protein interactions we have now applied it to investigate five important transcription factors, Mediator, DSIF, NELF, CTCF and GR.

## Materials and methods

### Expression and purification of DFF

The DFF40 and DFF45 sequences with inserted TEV sites were *Escherichia coli* codon-optimized (IDT codon optimization tool) and inserted bicistronically into an expression vector that directs the isopropyl β-d-1-thiogalactopyranoside (IPTG)-inducible expression under the control of a strong Ptac promoter with a His-Tag on the C-terminus of DFF40. NEBExpress^®^*I^q^* Competent *E. coli* cells (C3037I) were then transformed with the DFF expression vector, plated on ampicillin plates, and colonies were selected for growth in 5 ml of L broth (Invitrogen) supplemented with 100 μg/ml ampicillin with rotation at 37°C. After ∼8 h of growth, 1 ml of a single colony growth was then transferred to 1 l of ampicillin containing L broth and grown for another ∼4 h with rotation. Once the culture reached an optical density at 600 nm of 0.6 the cultures were induced with 0.1 mM IPTG, and incubated overnight at 18°C. The following morning bacterial cultures were spun down at 4500 × g at 4°C, resuspended in ∼15 ml of lysis buffer (1× PBS, 0.1% Triton X-100, 5 mM imidazole, 0.1% PMSF saturated in Isopropanol, 1 tablet of EDTA cOmplete protease tablet), and sonicated 5–6 times. Following sonication, a small aliquot of the crude lysate was taken and the remainder was salted up 150 mM sodium chloride and then spun in an ultracentrifuge at 244,000 × g at 4°C for 1 h. Afterwards, an aliquot of the supernatant was taken and the remainder was incubated with 2 ml Ni-NTA agarose beads for 1 h with rotation at 4°C and washed with high salt washes (10 mM Tris (pH 7.8), 150 mM sodium chloride, 35 mM imidazole, 1% PMSF) and low salt washes (10 mM HEPES (pH 7.6), 50 mM potassium chloride, 35 mM imidazole, 1% PMSF). Bound proteins were eluted with 10 mM HEPES (pH 7.6), 50 mm potassium chloride, 300 mM imidazole and 1% PMSF. After elution, samples taken at various stages of the purification were analyzed by SDS-PAGE and show strong enrichment of DFF40/45 heterodimers (Supplementary Figure S1A). The resulting product was then immediately spun for 15 min at 22 500 × g and the protein in the supernatant was FPLC purified on a Mono Q 5/50 GL column (Supplementary Figure S1B). Afterwards, DFF40/45 concentration was determined, and the optimal amount of TEV to add for DFF activation was tested. A time course of digestion at room temperature was carried out utilizing a TEV:DFF ratio of 1:3 or 1:100 (Supplementary Figure S1C). These ratios refer to the relative amount of DFF and TEV enzyme used based on total molecular weight (μg TEV: μg DFF) added directly to the Mono Q purified DFF. The 1:3 ratio yielded significantly faster digestion of DFF45 but led to precipitation of DFF with prolonged incubations. Additionally, comparison of room temperature digestions with 4°C digestions utilizing the 1:100 TEV:DFF ratio only affected the kinetics of digestion (Supplementary Figure S1C). Given these results, bulk DFF was then activated by TEV digestion at room temperature utilizing the TEV:DFF ratio of 1:100 and stored in aliquots at −80°C.

### Nuclei isolations and quantification

Details on nuclei isolation have been previously published (27). If indicated, crosslinking was performed with 1% formaldehyde for 10 minutes followed by addition of 3 M Tris (pH 7.8) to reach a final concentration of 1 M prior to the nuclear isolation for some samples. Additionally, SUP-B15 cells were treated with 1 μM dexamethasone (dex) for 1 h immediately before crosslinking and nuclei isolation. HeLa nuclei were grown to 90% confluency in DMEM (Gibco 11965092) supplemented with 10% FBS (Gibco 26140079) and SUP-B15 nuclei were grown in RPMI 1640 (Gibco 11875093) supplemented with 10% FBS (Gibco 26140079). Prior to freezing of isolated nuclei, three small aliquots of nuclei were removed for total DNA quantification. Aliquots were volumed up to 200 μl utilizing buffer containing 20 mM HEPES (pH 7.6), 5 mM magnesium acetate, 100 mM potassium acetate, 5 mM DTT and sonicated for 6 cycles of 20 s on and 40 s off utilizing a Qsonica Q800R3 sonicator at 40% amplitude. Next, the aliquots were treated with RNAse-A (Thermo-Scientific EN0531) for 30 min at 37°C and proteinase K (NEB 98107S) for 2 h at 55°C. The resulting 200 μl of sample were then extracted with an equal volume of phenol chloroform (1:1) and the soluble DNA was precipitated by addition of 3 volumes of 95% ethanol containing 0.5 M ammonium acetate. The resulting pellet was then washed with 70% ethanol and resuspended in water. The resulting DNA from each aliquot was quantified via nanodrop and the reading was averaged for each set of three aliquots. Next, assuming 7 pg of DNA per nucleus, the number of nuclei per volume was then back calculated for each nuclei stock.

### DFF digestions

The ability of DFF to digest DNA was tested using a standard protocol. A titration of DFF ranging from 3 ng to 100 ng DFF was utilized to digest 1 μg of a 6538 bp plasmid for 30 min in buffer containing 20 mM HEPES (pH 7.6), 5 mM magnesium acetate, 100 mM potassium acetate, and 5 mM DTT at 37°C. The resulting DNA was then analyzed via 1% agarose gel. With increasing concentration of DFF, the plasmid was progressively more digested yielding a range of products averaging approximately 75 bp with 100 ng of DFF (Supplementary Figure S1D, left). The activity of all future DFF preps was quantified with one unit of DFF (typically 100 ng) being the amount that can digest 1 μg of plasmid template to ∼75 bp in 30 min at 37°C. Additionally, we sought to test if DFF digestion was consistent between differing nuclei preps. After quantifying nuclei from SUP-B15, HeLa and two different HAP1 nuclei isolations, digestions were performed as follows. One million nuclei were incubated with either 150, 500 or 1500 ng of DFF for 30 min in buffer containing 20 mM HEPES (pH 7.6), 5 mM magnesium acetate, 100 mM potassium acetate, and 5 mM DTT at 37°C for 30 min (Supplementary Figure S1D, right). Following digestion, samples were treated with RNAse-A (Thermo-Scientific EN0531) for 30 min at 37°C and proteinase K (NEB 98107S) for 2 h at 55°C. The samples were then phenol chloroform extracted and the soluble DNA was precipitated by addition of 3 volumes of 95% ethanol containing 0.5 M ammonium acetate. The resulting pellet was then washed with 70% ethanol and resuspended in water. Incubation of nuclei with increasing levels of DFF yielded progressively more complete digestion of nucleosome ladders in multiple cell types from separate nuclei isolations. However, HAP1 nuclei from one preparation were less sensitive to DFF as compared to HeLa and SUP-B15 nuclei. Whether this is an actual phenotypic difference between these cell types or is indicative of a difference during nuclei isolation is unclear. Therefore, we recommend that for each new preparation of nuclei a time course and enzyme titration is carried out to ensure proper digestions. Moving forward, for each digestion of nuclei the number of nuclei and DFF utilized are reported.

### *In vitro* transcription

*In vitro* transcription reactions were based on an immobilized template transcription system described previously (28). PICs were assembled via incubation of a biotinylated HCMV promoter template immobilized to Dynabeads M280 Streptavidin (Invitrogen 11206D) with HeLa nuclear extract for 30 min in 20 mM HEPES (pH 7.6), 60 mM potassium acetate, 5 mM magnesium acetate, 1 mM DTT and 0.5 U/μl SUPERase•In. Transcription was initiated with the addition of a pulse mixture containing the nucleotides A, U and G at 1.5 mM (final concentration of 0.5 mM in the reaction) and α-[^32^P]-CTP for 30 s. Following the pulse, a single reaction was chased via addition of all NTPs. The remaining early elongation complexes (EECs) were washed twice with high salt wash containing 20 mM HEPES (pH 7.6), 1.6 M potassium acetate, 25 mM EDTA, 1 mM DTT, 0.1 mg/ml BSA, and 0.02% v/v Tween 20 and once with a low salt wash identical to the high salt wash but with 60 mM potassium acetate and without EDTA. The washed EECs were then separated into individual reactions and incubated in DFF buffer or mock buffer identical to the low salt wash buffer but containing 5 mM magnesium acetate and differing amounts of DFF. The total amount of DFF in each reaction was approximately 0, 0.7, 7, 70 and 700 ng of DFF. Following incubation for 30 min, the EECs were chased with the addition of NTPs to a final concentration of 1 mM for 10 min.

Nuclear walk-on reactions were conducted as done previously with slight modification (1,27). In short, 80 μl of nuclei (∼8 million nuclei) were pooled and diluted to 200 μl in either DFF containing buffer or mock buffer (20 mM HEPES (pH 7.6), 5 mM magnesium acetate, 100 mM potassium acetate, 5 mM DTT and 0.5 U/μl SUPERase•In). After 10 min of incubation DFF was inhibited by addition of EDTA, 40 μl was removed for a ‘total reaction’ from each condition. Next, the remaining nuclei were centrifuged and 40 μl of soluble material was removed. The remaining nuclei were sonicated for 2 s at 40% amplitude using Qsonica Q800R3 sonicator, centrifuged, and another 40 μl was removed. This was then repeated for 18 s of sonication. The supernatants were then utilized in traditional walk-on conditions for label incorporation that included Sarkosyl that would continue to inhibit DFF digestion under transcription conditions containing magnesium. Approximately 5 μg of DFF was utilized per 20 μl of nuclei.

### DFF-ChIP-Seq

For CTCF, NELF, DSIF and MED1 DFF-ChIP ∼20 million nuclei from HeLa cells were digested with ∼50 μg of DFF in 20 mM HEPES (pH 7.6), 5 mM magnesium acetate, 100 mM potassium acetate, 5 mM DTT, for 30 min at 37°C. For GR DFF-ChIP, ∼20 million nuclei from SUP-B15 nuclei were digested with 2 μg DFF in the same buffer for 30 min at 37°C since SUP-B15 nuclei were significantly more sensitive to DFF digestion. Prior to digestion, nuclei were pelleted (20 s in low speed Prism mini centrifuge) to remove the storage buffer and the nuclei were resuspended in the above buffer without DFF. Large scale digestions often resulted in reduced digestion efficiency possibly due to the settling of nuclei during digestion. Where possible, digestion in a single tube was capped at 10–20 million nuclei with a concentration of 50 000 nuclei per μl. Therefore, the digestion of 20 million SUP-B15 nuclei were done in two separate digestions in 200 μl each and pooled after DFF digestions were stopped with EDTA. To further prevent settling, about once every 5 min, the nuclei were resuspended with gentle flicking of the tube. Digestion was halted with the addition EDTA to a concentration two times that of magnesium and nuclei were subsequently split for individual IPs. Nuclei were lightly sonicated for 20 s at 40% amplitude using Qsonica Q800R3 sonicator and the supernatant was collected and brought up to 1 ml with solution containing 10 mM Tris (pH 7.5), 100 mM sodium chloride, 1 mM EDTA, and TritonX-100 such that the final concentration was 0.1%. The supernatants were precleared for 20 min over 20 ul of Protein A/G PLUS-Agarose beads (sc-2003). Afterwards, the supernatants were removed from beads and incubated with ∼5 μg of antibodies for CTCF (D31H2 XP^®^ Rabbit mAb #3418), NELF-A (A-20 sc-23599), DSIF (H-300 sc-28678), MED1 (A300-793A Bethyl Labs), or 5 μg antibody for GR (IA-1), a polyclonal rabbit antibody raised against human GR amino acids 84–112 (29), overnight at 4°C with rotation. Next, samples were incubated with 20 ul of Protein A/G agarose beads for 2 h at 4°C with rotation. The beads were then washed five times with 10 mM Tris (pH 7.5), 150 mM sodium chloride, 1 mM EDTA, and 0.1% TritonX-100 for 5 min per wash. Bound material was than eluted twice with 50 μl of 10 mM Tris (pH 7.5), 1% SDS, and 1 mM EDTA incubated at 65°C for 5 min. Eluted material was subsequently treated with 20 μg RNAse A for 30 min at 37°C and then 40 μg of Proteinase K for 2 h at 65°C. The resulting DNA was phenol chloroform extracted. TruSeq adapters were then attached that had an 8 bp unique molecular identifier (UMI) immediately downstream of the index utilizing NEBNext^®^ Ultra™ II DNA Library Prep Kit for Illumina^®^ kit which includes DNA end repair steps (see Supplementary Data File). Test amplifications were performed on each library to determine the number of cycles necessary to obtain enough library material for sequencing. Full-scale amplification was then performed, fragments were quantified using an Agilent Bioanalyzer 2100, and then libraries were pooled and size selected from 135–1000 bp using a BluePippin. Prior to submission, proper size selection was confirmed with reanalysis using the Agilent Bioanalyzer. Libraries were sequenced on a NovaSeq 6000 in paired-end 50 bp format and converted to fastq using bcl2fastq2 (version 2.2) by the Iowa Institute of Human Genetics.

### ChIP-Seq

Three different protocols (Exp 1–3) were utilized for generation of GR ChIP-Seq data with differing crosslinking conditions, quenching buffers, and shearing times. For all workups the B cell acute lymphoblastic leukemia cell line SUP-B15 was grown in RPMI (Gibco,11875093) + 10% fetal bovine serum (Gibco, A5256701). The afternoon before, cells were counted, resuspended in fresh medium, and aliquoted into 6-well plates at 5 million cells per well. In the morning, cells were treated with 1 μM dex (Sigma, D4902-1g) or vehicle (0.1% ethanol) (Decon Laboratories, 2716) control for 1 h. For Exp 1, cells were crosslinked with 0.5% fresh formaldehyde (Thermo Scientific, 28906) for 3 min and the crosslinking reaction was treated with 125 mM glycine (RPI, G36050-1000.0). For Exp 2, cells were crosslinked with 1% fresh formaldehyde for 6 min and quenched with 750 mM Tris (pH 8.0) (RPI, T60050-1000.0). Finally, for Exp 3, cells were crosslinked with 1% fresh formaldehyde for 10 min and quenched with 750 mM Tris (pH 8.0). All crosslinked cells were washed in PBS (Hyclone, SH30028.LS) and then sheared using truChIP Chromatin Shearing Kit (Covaris, 520154) and a Covaris E220 focused ultrasonicator. Shearing time was 8 min for Exp 1 and 10 min for Exp 2 and Exp 3. GR was immunoprecipitated with 5 μg of the N499 antibody (a generous gift from Keith Yamamoto), and chromatin was isolated with magnetic Protein G beads (Invitrogen, 10004D). Crosslinks were reversed and proteins were removed by incubation with Proteinase K (New England Biolabs, P8107S) for 2 h at 65°C, and DNA was purified by phenol chloroform extraction. For each ChIP-Seq protocol, the resulting DNA was split equally three ways and prepped into libraries with three different kits (Nugen Ovation Ultralow-0330-32, BIOO Nextflex-5143-01 or Nextflex Rapid-5144-02), quantified, and sequenced (Illumina HiSeq 2000) in paired-end 50 bp format to a depth of ∼30 million each. Initial processing of the data indicated that differing methods gave similar results. Therefore, reads were combined for dex-treated samples for many analyses. Finally, two replicates of ChIP-Seq were performed on B1 cells targeting GR with the IA-1 antibody utilized in DFF-ChIP and worked up with the same crosslinking conditions as Exp1. Both replicates were conducted with and without dexamethasone treatment. Sequencing was done on a HiSeq 2000 Illumina sequencer.

### Processing of DFF-ChIP-Seq, ChIP-Seq and CUT&RUN

A custom Snakemake pipeline (https://doi.org/10.5281/zenodo.10079297) was used to process fastq files into bigWigs. Briefly, fastq files were trimmed of adapter sequences with trim_galore v0.6 (https://github.com/FelixKrueger/TrimGalore) with length parameter of 18 to 1000 bp with command –paired –adapter AGATCGGAAGAGCACACGTCTGAACTCCAGTCA –adapter2 AGATCGGAAGAGCGTCGTGTAGGGAAAGAGTGT –quality 0. For ChIP-Seq trimming, the program's adapter auto-detection was utilized. Sequences were aligned as follows with bowtie v1.2.3 (30): B1 GR ChIP-Seq and CTCF CUT&RUN to the human genome (UCSC assembly hg38), GR DFF-ChIP to a concatenated hg38 and mm39 assembly due to the usage of 4T1 nuclei spike-ins, and all other datasets to hg38 with modification (UCSC assembly hg38 with all unplaced and unlocalized contigs removed and 237683 bp of HCMV TB40e concatenated). Alignment was done with command –minins 18 –maxins 1000 –fr –best –allow-contain. Eight nt UMIs were present in the index reads for DFF-ChIP experiments (R2) and were used for dedupping (https://doi.org/10.5281/zenodo.10041794). Total read depth and alignment statistics are present in the Supplementary Data File. Dedupped files were converted into bedGraphs with bedtools genomecov v2.27.1 (31) with command bedtools bamtobed -I input.bam -bedpe -mate1. We then generated bigwig tracks with the bedGraphToBigWig program from kentUtils (https://github.com/ENCODE-DCC/kentUtils/tree/master/bin/linux.x86_64). All DFF-ChIP experiments were performed in duplicates and Pearson correlations were calculated after dividing the genome into 10 kb bins and summing the reads for each bin (Supplementary Figure S2).

### ChIP-Seq-Peak analysis

ChIP-Seq-Peak (https://doi.org/10.5281/zenodo.10914111) is a program that takes as an input ChIP-Seq data and first generates a file with 25 bp bins. Anytime a bin is not a part of an 8 consecutive bin chain (consecutive 200 bp) they are removed. Next, bins that have higher signal than both the immediately adjacent downstream and upstream bins are considered potential peaks. This creates a large list of peaks that has many false positives. To remove these false peaks, the minimal bin between two chosen bins is first found. Then, if the height of the minimum bin is <60% of the smaller of the two bins, the two bins are well-separated and utilized for peak calling. If not, the second peak is removed and the program searches for the next minimum between the original bin and the next bin it finds greater than its adjacent bins. This continues until peaks meet the criteria or if the distance between the two peaks is >200. If so, the two bins are utilized for peak calling. Next, to best characterize the called peaks, a normal distribution is generated from the raw data utilizing peak height, position of the center of the peak, and assuming a 400 bp width. The program optimizes these parameters around the called peak and then draws a 10 bp line over the peak of this normal distribution with a height equal to the number of reads in the peak. For our usage on ChIP-Seq data targeting GR, we first library size normalized and subtracted GR-ChIP-Seq dex and GR-ChIP-Seq nodex datasets to eliminate regions of background signal. Next, ChIP-Seq peak was run on the subtracted data and peaks >75 reads were utilized for further analysis. Some centromeric regions yielded peaks that were still >75 in height even after background subtraction and were blocklisted. The blocklisted regions were as follows: chr1:125037718–143553145, chr4:49056148–49737993, chr10:39350927–42213196, chr16:38069295–46485764, chr21:8144873–8557778 and chrY:26450075–57055992.

### DFF-ChIP-Seq-Peak (DFF-CSP)

DFF-CSP (https://zenodo.org/doi/10.5281/zenodo.10891329) takes bedfiles, determines the center of each fragment, and calls clusters of fragment centers across the genome and is useful for identification of sites of occupancy of sequence specific transcription factors. These clusters are referred to as center clusters (CCs) and these are used to calculate peaks of occupancy. Based upon the data used one may modify the CC window size or CC read depth. CC window size is a parameter influenced by how focused or well positioned targeted peaks are with stronger positioning requiring smaller CC window sizes. CC read depth is a threshold value that determines how many centers must be in a CC for it to be called. Running DFF-CSP results in a tab-delimited text file which contains information about the called and non-overlapping CCs. For IRF analysis with DFF-CSP regions were blocklisted as follows: chr5:49600783–49670029, chr10:41838938–42110985, chr15:19773761–19792684, chr20:26560426–28500888, chrY:56672149–57217277.

### Heatmaps and fragMaps

Heatmap.py (https://doi.org/10.5281/zenodo.8384131) was used to create heatmaps. Sorts for heatmaps are described in the text. FragMap.py (https://doi.org/10.5281/zenodo.8384148) was used to make fragMaps displaying the average distribution and position for DFF-ChIP data.

### Motif discovery

DFF-CSP was utilized to find CCs in the native and crosslinked CTCF DFF-ChIP datasets resulting in 18875 and 32905 CCs, respectively. The native CCs were expanded from 25 to 125 bp and the 100 bp crosslinked CCs were not expanded prior to analysis with MEME (32) which resulted in 18,335 native and 32216 crosslinked CCs containing the 15 bp CTCF. To compare DFF-ChIP to CUT&RUN, all 1,048,505 CTCF motif occurrences were found across the genome utilizing the CTCF motif discovered with native DFF-ChIP and the FIMO functionality of the MEME Suite (32). Any of these motif sites with more than 200 reads were chosen for further analysis resulting in 19,184 CTCF-CUT&RUN sites, 16,056 native CTCF DFF-ChIP sites, and 27,442 crosslinked DFF-ChIP sites. For GR site analysis, sequences ±100 bp from the center of the 14 187 called peaks found with ChIP-Seq-Peak were utilized as inputs into MEME resulting in 9,772 peaks containing the consensus GR motif. For IRF site analysis, DFF-CSP was utilized to find CCs in the native GR DFF-ChIP datasets resulting in 10,506 CCs and MEME analysis resulted in 6,569 peaks containing the IRF motif. The parameters utilized for each DFF-CSP run are discussed in further detail in the results section.

## Results

### Optimization of DFF-ChIP and library preparation

**Figure 1.**
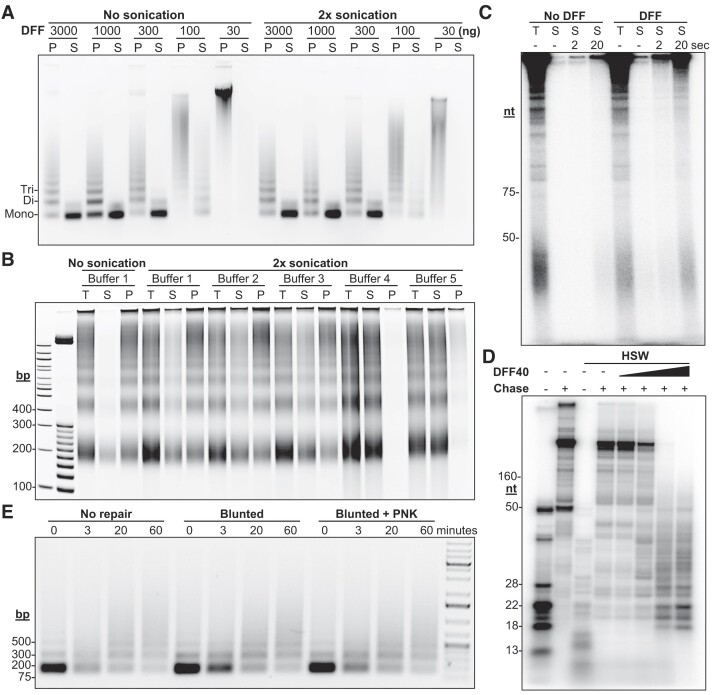
Applicability of DFF to ChIP protocols. (**A**) Digestion of 2 million native HAP1 nuclei with the indicated amounts of DFF prior to separation of soluble (S) and insoluble pellet (P) material via centrifugation or prior to sonication and separation. Resulting products were run on an agarose gel and stained with ethidium bromide. (**B**) 40 million crosslinked HAP1 cell nuclei were digested with 10 μg of DFF for 2 h. The reaction was split into 6 tubes which were then supplemented with buffer to generate the differing conditions to alter solubility of DNA. All buffers had 25 mM Tris (pH 7.8) and 1 mM EDTA and the following additions: 100 mM sodium chloride (1), 100 mM sodium chloride and 0.1% TritonX (2), 150 mM sodium chloride, 1% TritonX, and 0.25% sodium deoxycholate (3), 150 mM sodium chloride and 0.2% Sarkosyl (4), or 150 mM sodium chloride and 0.1% SDS (5). After buffer supplementation samples were lightly sonicated prior to separation via centrifugation. The resulting products were run on a native polyacrylamide gel and stained with ethidium bromide. Samples are total (T), soluble (S) and insoluble pellet (P). (**C**) HAP1 nuclei were incubated alone or digested with DFF for 30 min at 37°C. Following digestion, transcripts were labeled via incubation with α-^32^P-CTP. Next, reactions were either sonicated lightly for 2 or 20 s or immediately spun to isolate soluble materials (S) for comparison against the total reaction (T) using a denaturing polyacrylamide gel. (**D**) An immobilized DNA template was incubated with HNE for 30 min and pulsed for 30 s with limiting α-^32^P-CTP. The resultant EECs were then washed and treated with a titration of DFF for 10 min. Next, EECs were subsequently chased for 1 minute. The resulting products were run on a denaturing polyacrylamide gel and radioactivity imaged. As a note, the presence of magnesium in the mock buffer allowed for slight backtracking of the paused transcripts in the mock buffer incubated sample. (**E**) HeLa nuclei were digested with DFF to primarily mono-nucleosomes and DNA isolated. The DNA was then subjected to blunting by T4 DNA polymerase, blunting and an additional end repair utilizing T4 Polynucleotide Kinase (PNK), or left unrepaired. The resulting DNA was then incubated with T4 DNA ligase for differing times and run on an agarose gel and stained.

DFF has been proven useful in determining the global occupancy of transcription machinery, chromatin, and how the two interact (1,18,24–26). Here we wanted to provide further information on the purification of DFF, optimization of nuclei digestion and solubilization of protein:DNA complexes after digestion. Details of the purification of active DFF, quantification of DFF activity on naked DNA and application to nuclei digestion are provided in the Materials and Methods section and Supplementary Figure S1. To more fully characterize the solubilization of DFF digestion products, HAP1 nuclei were isolated and digested under native conditions with a range of DFF concentrations without and with mild sonication followed by separation of soluble and insoluble material via centrifugation (Figure 1A). Most mono-nucleosomes and a fraction of the di-nucleosomes sized fragments were released and sonication did not strongly influence this. The smaller size of released mono-nucleosome fragments suggests that these nucleosomes are more susceptible to DFF digestion, and the smaller size of di-nucleosomes fragments suggests that these nucleosomes are also more closely packed. Because crosslinking is normally used in standard ChIP, we also determined the solubility of DFF digested DNA from crosslinked nuclei (Figure 1B). After DFF digestion, crosslinked nuclei without or with light sonication were separated into soluble and insoluble fractions by centrifugation in the presence of Buffer 1. Under both conditions, only a fraction of total material was released, but sonication improved this fraction. In an attempt to increase release, digested nuclei were also sonicated in varying buffers with increasing detergent severity (Buffer 2: 0.1% TritonX-100, Buffer 3: 1% TritonX-100 and 0.25% sodium deoxycholate, Buffer 4: 0.2% Sarkosyl, Buffer 5: 0.1% SDS). Buffers 2 and 3 mildly improved solubilization. Buffers 4 and 5 resulted in complete solubilization, but because of the likely negative impact of the harsh detergent conditions on immunoprecipitation, we chose to use Buffer 2 with light sonication for both native and crosslinked DFF-ChIP experiments.

Because DFF-ChIP has been used to study active transcription complexes (1,18,24–26) we wanted to optimize the solubilization of those complexes. A modification of our nuclear walk-on method (27) was performed as follows. Nuclei were incubated with or without DFF for 10 min at 37°C, then EDTA was added to inhibit DFF, and samples were taken either immediately to represent the total reaction or from soluble material after 0, 2 or 20 s of sonication. Each sample was analyzed in a walk-on reaction by incubation with ^32^P-CTP for 10 min (Figure 1C). During this time, DFF would remain inactive due to the presence of Sarkosyl. Without digestion there is essentially no label incorporation in the soluble fractions and only a small amount of incorporation is detected with 20 s of sonication. With DFF digestion, label incorporation of the soluble material is improved, and 2 s of sonication shows slight improvement. With 20 s of sonication after DFF digestion, most of the label incorporation is recovered indicating release of nearly all engaged Pol II. However, the total amount of label incorporation was slightly reduced after DFF digestion. To determine if this effect was due to disruption of engaged Pol II, an *in vitro* transcription system with HeLa nuclear extract and an immobilized DNA template was used to isolate Pol II elongation complexes. These were then digested with increasing amounts of DFF and then chased. All levels of DFF digestion resulted in EECs transcribing further down the template with EECs subjected to the highest level of DFF digestion halting early about 20 nt downstream of the initial stop sites (Figure 1D). The two highest levels of DFF digestion differed by an order of magnitude but still yielded the same pattern with retained signal intensities. This indicates that even a great excess of DFF yielding limit digests does not disrupt engaged Pol II.

Prior studies on DFF (33) indicate that it cuts both strands of DNA to leave undamaged blunt ends, suggesting that no repair is needed in subsequent ligations. To verify this, we isolated DNA from nuclei digested with DFF down to mono-nucleosome sized fragments and then compared the ligation efficiency of isolated DNA before and after end-repair by blunting or by blunting with end phosphorylation using PNK. When the DNAs were incubated with T4 DNA ligase for increasing amounts of time more and more of DFF generated nucleosome sized fragments shifted from primarily mono-nucleosome in size to various di-, tri-, and tetra-nucleosome sizes regardless of end repair over the time course of ligation indicating that DFF leaves ends suitable for ligation reactions (Figure 1E). This result also suggests that DFF may have applications for different chromatin capture protocols that rely on such ligations.

### DFF-ChIP better resolves transcription complexes in comparison to other methods

**Figure 2.**
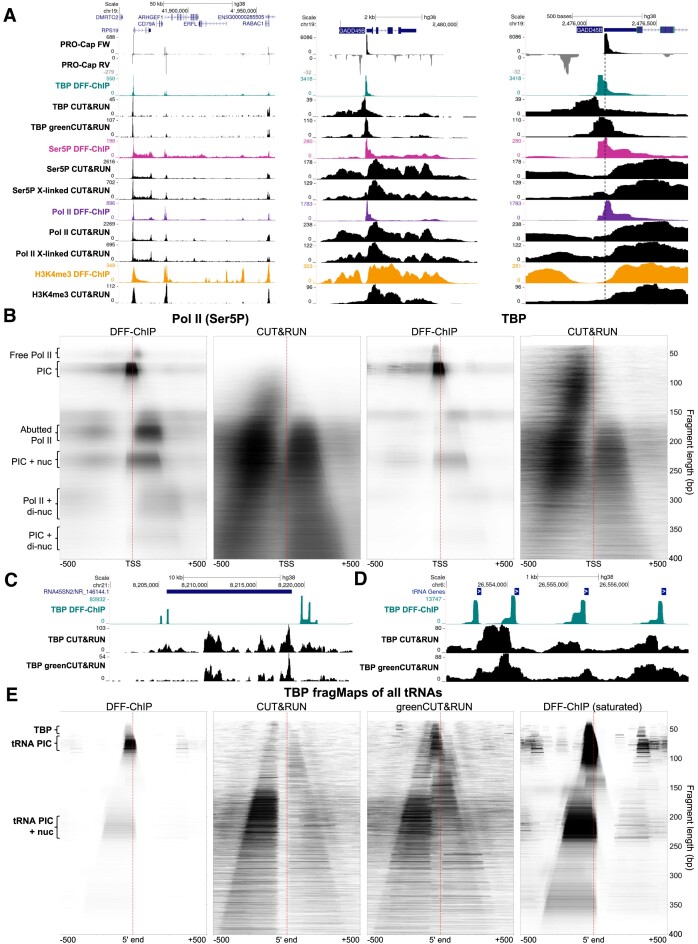
Comparison of DFF-ChIP to CUT&RUN. (**A**) UCSC genome browser tracks showing HFF PRO-Cap dataset (GSE113394), CUT&RUN for TBP (GSE163049), Ser5P (GSE155666), Pol II (GSE155666) and H3K4me3 (GSE156787) compared to DFF-ChIP datasets (GSE185763). DFF-ChIP and CUT&RUN were not performed utilizing the same antibodies. The first region approximately depicts chr19:41850000–42000000, the second region chr19:2474000- 2481000, and the final region chr19:2475500–2476750. In the right-most region, the primary TSS for GADD45B is demarcated by a dotted line for ease of alignment. (**B**) FragMaps comparing DFF-ChIP targeting Ser5P and TBP to CUT&RUN targeting the same factors. FragMaps are centered on the MaxTSS of 11,229 genes expressed in HFF cells discovered utlizing the truQuant on the PRO-Cap dataset. (**C**) UCSC genome browser tracks of DFF-ChIP, CUT&RUN, and greenCUT&RUN targeting TBP showing a 45s preRNA region. (**D**) UCSC genome browser tracks of DFF-ChIP, CUT&RUN, and greenCUT&RUN targeting TBP showing four tRNA genes. (**E**) FragMaps comparing DFF-ChIP targeting TBP to CUT&RUN (GSE163049) and greenCUT&RUN (SRP278136) targeting TBP. FragMaps are centered on the mature 5′ ends of 534 tRNA genes.

Our previous work utilizing DFF-ChIP in contact inhibited, primary human foreskin fibroblasts (HFFs) showed unprecedented levels of precision when examining transcription complexes with near-base pair resolution of PICs, engaged Pol II, and the interactions of those complexes with the local chromatin (18). We compared our results with those achieved with CUT&RUN (34–37) in various cell types for H3K4me3 (FaDu), TBP (K562), the Ser5P modification on the CTD of Pol II (A549), and Pol II itself (A549). Additionally, greenCUT&RUN targeting TBP (HeLa) (35) was analyzed, which utilized a GFP nanobody/MNase fusion protein rather than a specific antibody coupled with a protein-A MNase fusion protein for digestion. Tracks of multiple genes from the DFF-ChIP and CUT&RUN datasets both show enrichment of promoters and gene bodies well over background (Figure 2A, left). However, examination of single genes and promoters shows significant differences (Figure 2A, right). TBP DFF-ChIP over the GADD45B promoter clearly shows signals corresponding to TBP alone, the full PIC, and PIC interacting with the downstream nucleosome that we have previously described (18). TBP CUT&RUN tracks show a wider distribution compared to DFF-ChIP with standard CUT&RUN showing a nucleosome sized peak completely distinct from the entire PIC that DFF-ChIP recovers. GreenCUT&RUN does recover some of the PIC signal but is less discrete than DFF-ChIP. Ser5P DFF-ChIP shows signal from the PIC, promoter proximal paused Pol II, and elongating Pol II throughout the gene and downstream of the poly(A) site, as expected. The two Ser5P CUT&RUN datasets, with and without crosslinking, both fail to show any PIC signal and significantly reduced paused Pol II signal. Pol II DFF-ChIP, like Ser5P, recovers PIC signal but has significant enrichment for paused Pol II. These discrete signals are not found in Pol II CUT&RUN and the data that is recovered is nearly identical to Ser5P CUT&RUN. Finally, H3K4me3 data from both DFF-ChIP and CUT&RUN show positioning of the nucleosomes surrounding the promoter region. The pattern of H3K4me3 occupancy is more similar between the two methods compared to the analysis of transcription complexes.

To visualize the size and position of protected fragments, fragMaps were generated utilizing the Pol II (Ser5P) and TBP datasets from CUT&RUN and DFF-ChIP. A fragMap is a 2D heatmap that depicts fragment position on the x-axis and fragment size on the y-axis, as described previously (18). Here, fragMaps are centered upon 12,229 TSSs discovered utilizing truQuant, a program that identifies the MaxTSS of expressed genes, on high-quality PRO-Cap data from HFFs. Pol II (Ser5P) fragMaps utilizing the DFF-ChIP data show multiple discrete features that correspond to both PICs and engaged Pol II as well as their interactions with downstream nucleosomes (Figure 2B). However, CUT&RUN fails to reveal any of these discrete features, only showing nucleosome sized protections with enrichment upstream and downstream of the TSS. TBP DFF-ChIP enables visualization of the PIC, the PIC plus the downstream nucleosome, and TBP bound only to the TBP binding motif. TBP CUT&RUN does show enrichment of small fragments, but these are exclusively upstream of the TSS and far more varied in size compared to small fragments recovered with DFF-ChIP. Overall, DFF-ChIP recovers transcription complexes which are lost in CUT&RUN, while CUT&RUN predominately yields nucleosomes in the region.

Previously, we have reported that TBP DFF-ChIP recovers strong signals on genes driven by both Pol I and Pol III, which both utilize TBP during initiation (24). Pol I is responsible for the synthesis of ribosomal RNA (rRNA) and transcribes the 45S precursor RNA. The 45S rRNA sequence is repeated many times across the genome and at many of these loci DFF-ChIP identifies specific features. TBP-DFF ChIP yields signal at the primary promoter for 45S and the upstream spacer promoter, but also signal at the end of 45S at sites closely related to TTF1 termination sites as we have previously shown (Figure 2C) (24). The rRNA repeats are strongly transcribed and the amount of TBP signal obtained at these sites is correspondingly high. However, CUT&RUN shows almost no specific signal at these sites. Pol III is responsible for transcribing tRNAs, the 5S rRNA, and various other small RNAs. We have previously described the TBP signals found at these sites (24). The four tRNAs shown in Figure 2D exemplify the standard tRNA Pol III PIC which frequently has a well-positioned upstream nucleosome. The CUT&RUN data do not resemble the DFF-ChIP data in that the transcription complexes are missing. FragMap analysis of 534 tRNAs shows that TBP DFF-ChIP recovers the PIC and the PIC associated with the upstream nucleosome (Figure 2E). There is no PIC feature seen in the standard CUT&RUN fragMap. Instead, there is mainly an upstream nucleosome recovered that does not overlap with the PIC. Showing improvement over standard CUT&RUN, greenCUT&RUN does show a small amount of PIC protection. Interestingly, the PIC is instead connected to apparently sub-nucleosomal fragments from the downstream nucleosome. A similarly sized feature is detectable in DFF-ChIP but at a very low level relative to the other features.

### DFF-ChIP recovers transcription complexes associated with MED1, DSIF and NELF

**Figure 3.**
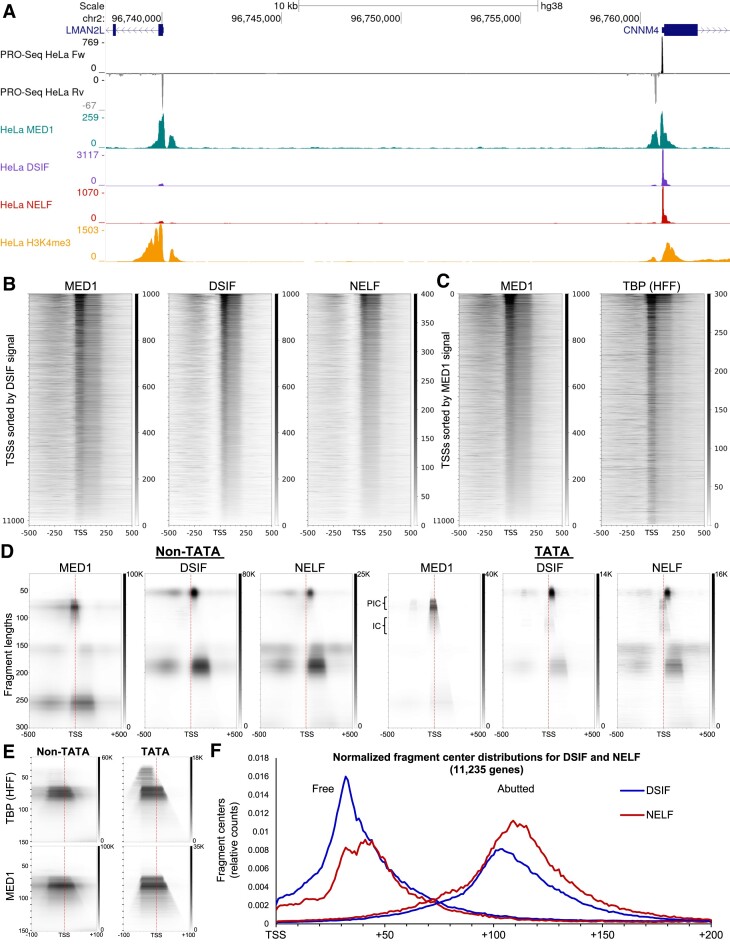
DFF-ChIP allows targeting of non-DNA bound factors. (**A**) UCSC genome browser tracks showing HeLa PRO-Seq (GSE100742) and DFF-ChIP targeng MED1, DSIF, NELF, and H3K4me3 (GSE185763) on two promoters. (**B**) MED1, DSIF, and NELF DFF-ChIP heatmaps of 11235 HeLa expressed genes sorted by DSIF signal from -50 to + 250. (**C**) MED1 DFF-ChIP and TBP DFFChIP (GSE185763) heatmaps of 11,235 HeLa expressed genes sorted by MED1 signal from -50 to + 250. (**D**) FragMaps of Non-TATA (10380) and TATA (500) genes from the MED1, DSIF, and NELF datasets. (**E**) Narrow range fragMaps of TBP from HFF cells and MED1 over Non-TATA and TATA genes. (**F**) Fragment center distribution of either paused (40–60 bp) or abutted (170–210 bp) sized fragments from DSIF (blue) or NELF (red) DFF-ChIP datasets relative to 11235 HeLa TSSs (see Supplementary Data File).

To determine if DFF-ChIP could be used to examine the occupancy of targets that do not directly interact with DNA, antibodies to subunits of Mediator, DSIF and NELF were used on DFF digested, native HeLa nuclei. All three factors showed strong enrichment over promoter regions (Figure 3A, Supplementary Figure S3A). Although levels of DSIF and NELF consistently matched the levels of transcription, levels of MED1 varied. As shown by the LMAN2L and CNNM4 promoter, both DSIF and NELF DFF-ChIP directly reflect the 11-fold difference in PRO-Seq signal whereas MED1 is present at similar levels on both promoters (Figure 3A). The amounts of MED1 also does not correlate with the amount of transcription over the sense and divergent transcription complexes for both promoters shown. This discrepancy prompted us to investigate how MED1, DSIF and NELF correlated on a wide range of promoters. To do so truQuant was utilized to find the MaxTSS of 11235 genes expressed in HeLa cells and heatmaps were generated sorted by the DSIF signal −50 to +250 from the MaxTSS. MED1 resulted in PIC and PIC nucleosome signals and DSIF and NELF resulted in both free and abutted Pol II signals (Figure 3B). Quantitatively, the DSIF and NELF signals correlated best with each other and MED1 correlated less well with DSIF and NELF (Pearson correlations, *r*: DSIF to NELF: 0.8, DSIF to MED1: 0.65, MED1 to NELF: 0.35). Heatmaps for HeLa MED1 DFF-ChIP and HFF TBP DFF-ChIP were sorted by the MED1 signal to compare PICs identified used MED1 and TBP (Figure 3C). The resulting heatmaps were very similar confirming that MED1 associates with PICs. Based on the example genes in Figure 3A and others we wondered if the MED1 signal might correlate with the amount of productive elongation experienced from promoters. HeLa PRO-Seq data (38) were used to calculate the ratio of paused Pol II with and without flavopiridol, the P-TEFb inhibitor that blocks the transition into productive elongation; the higher the flavo/control ratio the more productive elongation. When promoters were sorted from high to low there was no visual correlation of the MED1 signal or the NELF and DSIF signals (Supplementary Figure S3B). However, as expected the levels of NELF were lower for the genes with the most productive elongation. Evidently, the level of Mediator does not have a major influence on the fraction of paused Pol II that enters productive elongation.

The initial generation of fragMaps for the MED1, NELF and DSIF datasets resulted in some unexpected features overlapping the TSS. To determine if promoter type influenced these features genes were first separated into Non-TATA (10,380) and TATA (856) containing gene groups based on the presence of an appropriately positioned TBP-dependent motif identified earlier (39). FragMaps were then generated from the Non-TATA containing genes (Figure 3D, left) and the top 500 TATA (based on motif match) containing genes (Figure 3D, right). MED1 fragMaps indicate that PICs and PIC nucleosome features are recovered in both the non-TATA genes and TATA genes, but the PIC nucleosome is more prevalent on non-TATA genes. DSIF and NELF fragMaps both show free and abutted Pol II features on TATA and Non-TATA genes and, as seen for MED1, there is more nucleosome associated signal on the non-TATA genes. Surprisingly, the PIC feature showed up in the NELF fragMap and to a lesser extent in the DSIF fragMap for TATA genes. This is likely due to background signal for the PIC which is, except for nucleosomes, the most prevalent feature in promoter regions. These nucleosomes are also seen as background signal, especially for the NELF dataset. Interestingly, on the TATA genes both NELF and DSIF display a new feature that overlaps both the PIC region and paused Pol II. This 100 to 130 bp feature containing initiated complexes can also be seen in the MED1 TATA fragMap and to a lesser degree in the MED1 non-TATA fragMap. To examine what MED1 was interacting with in more detail, fragMaps ±100 bp around the TSS with fragments lengths up to 150 bp were generated for MED1 and TBP (HFF) on both Non-TATA and TATA genes (Figure 3E). As seen before (18), TBP gave a PIC feature (65–85 bp) on Non-TATA and TATA genes and a ‘free TBP’ feature (35–55 bp) over the TBP-dependent motif on TATA genes. MED1, which is known to interact with Pol II (9), did not interact with the free TBP feature. The PIC feature in the MED1 dataset emphasized the longer fragments (78–85 bp) which are extended in the direction of transcription and likely represent initiated complexes with short transcripts that have not released contact with the initiation machinery.

Mediator, DSIF and NELF DFF-ChIPs were also performed on crosslinked HeLa nuclei. DSIF and NELF gave similar patterns of occupancy with and without crosslinking (Supplementary Figure S3A). However, MED1 was very different with much non-specific background signal. FragMaps generated for crosslinked DSIF and NELF for non-TATA and TATA gene sets gave similar free and abutted Pol II features found in the native datasets with the expected loss of precision in fragment sizes (Supplementary Figure S3C). The MED1 crosslinked fragMaps surprisingly lacked PIC features, but instead had a distribution of various sized fragments in the nucleosome free region upstream of the TSS (Supplementary Figure S3C). This region is where a variety of sequence-specific transcription factors would reside that could interact with Mediator. Those factors might not be stably bound under native conditions which would explain why that feature is not prevalent in the native MED1 fragMap. It is not clear why the PIC is not well detected in the crosslinked MED1 fragMap.

Visual inspection of fragMaps and genome browser tracks suggested a slight downstream shift of the free and abutted Pol II features associated with NELF compared to the Pol II features associated with DSIF. To investigate this directly, fragment centers of all free Pol II fragments (40–60 bp) and abutted Pol II fragments (170–210 bp) were found in the region from +1 to +200 relative to all 11235 TSSs from the DSIF and NELF datasets and total signals were normalized. Plotting of these centers indicates that free Pol II complexes containing NELF are less represented relative to DSIF containing complexes prior to position +42 (Figure 3F). This result likely reflects the sequential recruitment of DSIF followed by NELF. Interestingly, abutted Pol II fragment centers detected with NELF are shifted ∼10 bp downstream relative to DSIF abutted Pol II fragment centers. However, since every NELF containing complex should have DSIF associated (40) one would expect DSIF to also show this shift. To explain this, we favor an argument in which NELF only represents a fraction of paused Pol II complexes and that these complexes may remain in the pause region longer.

### DFF-ChIP of human transcription factor CTCF precisely determines sites of occupancy

To expand the range of DFF-ChIP beyond transcription complexes, it was performed on native and crosslinked HeLa nuclei targeting the well-characterized transcription factor CCCTC-binding factor (CTCF), which binds strongly to its consensus binding site (*K*_d_ = 1 × 10^−11^ M) (41). CTCF is key to creating and maintaining large-scale chromatin conformations via anchoring chromatin loops (42). These loops isolate regions of the genome leading to preferential self-association regions called topologically associated domains (TAD). CTCF achieves this function via the blockage of hypothetical cohesin loop extrusion (43,44). To analyze how DFF-ChIP performs relative to CUT&RUN for CTCF, previously published (20) CTCF CUT&RUN datasets from K562 cells were combined and analyzed.

**Figure 4.**
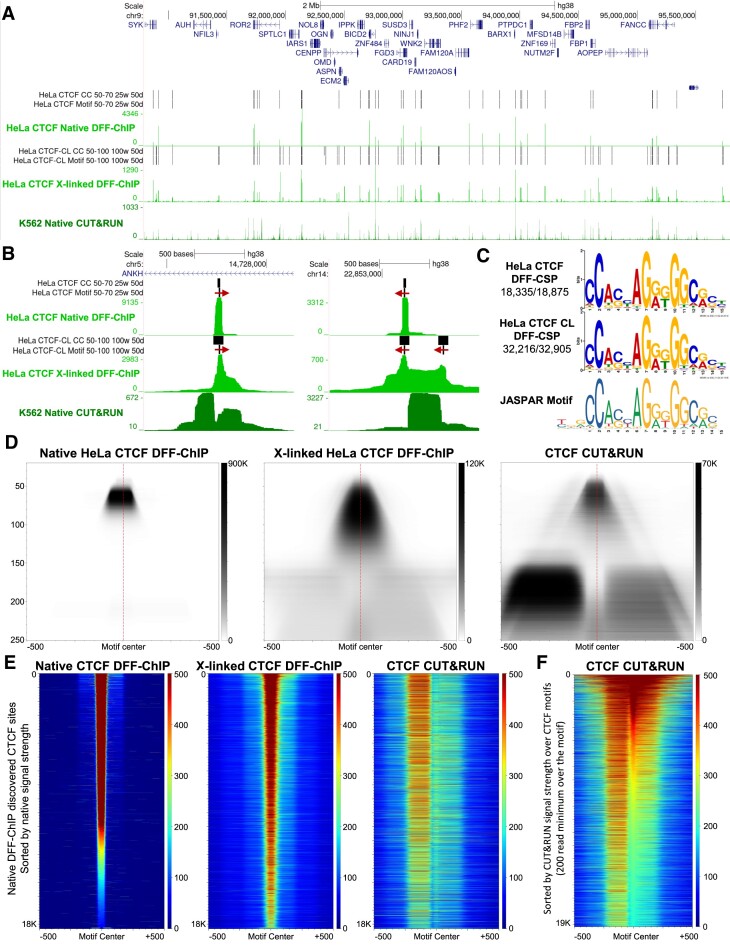
DFF-ChIP targeting CTCF. (**A**) UCSC genome browser tracks showing native CTCF DFF-ChIP, crosslinked CTCF DFF-ChIP, and CTCF CUT&RUN (GSE84474). Every called center cluster (CC) and every CC with a CTCF motif are shown for two different runs of DFF-CSP on either the native or crosslinked CTCF DFF-ChIP datasets. (**B**) UCSC genome browser tracks around two CTCF sites showing native and crosslinked CTCF DFF-ChIP and CUT&RUN. The direction of the motif is indicated by a red arrow. (**C**) CTCF motifs discovered by utilizing the MEME motif discovery tool on DFF-CSP outputs for native and crosslinked CTCF DFF-ChIP. Below is the CTCF motif from the JASPAR database (MA0139.1). (**D**) FragMaps comparing CTCF DFF-ChIP to CTCF CUT&RUN. FragMaps are centered on the 15 bp motif oriented in the direction of the motif in (**C**) of the 18335 sites identified in the native CTCF DFF-ChIP dataset. (**E**) Heatmaps centered on the 15 bp motif of 18335 sites identified in the native CTCF dataset sorted by over the CTCF motif from the native dataset. (**F**) Heatmap centered on the 15 bp motif from the CTCF CUT&RUN dataset. Sites were discovered utilizing motifs identified with the MEME suite FIMO tool to identify every occurrence of the CTCF motif across the genome. Of these motifs, those with 200 reads covering the 15 bp motif were selected and sorted by coverage over the CTCF motif in the CUT&RUN dataset (*n* = 19 184).

CTCF DFF-ChIP shows very strong signal at multitudes of locations across the human genome. This is especially true with native DFF-ChIP which has a dynamic range spanning three orders of magnitude (Figure 4A). In comparison to the native condition, crosslinked DFF-ChIP recovers more CTCF sites but with a less dynamic range and a differing pattern of peak heights. In support of DFF-ChIP, published CUT&RUN datasets targeting CTCF recovered many of the same sites as DFF-ChIP. However, the advantage DFF-ChIP provides becomes clear upon closer inspection of individual CTCF sites. Almost every region recovered with native and crosslinked DFF-ChIP shows a protection of approximately 70 bp directly over CTCF motifs (Figure 4B). Additionally, there is a slight preference for nucleosome recovery that favors the right-hand side of the motif and this preference is emphasized in the crosslinking condition. Different sites of CTCF occupancy from DFF-ChIP have differing amount of this interaction and close packed CTCF sites greatly emphasize these nucleosome features (Figure 4B, right). In comparison, CUT&RUN primarily releases the nucleosome that is opposite of the direction of the CTCF motif with only a small fraction of recovered DNA being due to CTCF protection alone. The asymmetric release of local nucleosomes has previously been shown (20). This limits the ability of CUT&RUN to accurately identify the binding site. Furthermore, the released nucleosomes are not associated with CTCF.

Deeper analysis of CTCF DFF-ChIP datasets began with peak identification and underlying sequence analysis of those peaks. To accurately identify the sites of occupancy, DFF-ChIP-Seq peak (DFF-CSP) was utilized (25). For native CTCF-DFF-ChIP, fragments of 50–75 bp in size were chosen, a scanning window of 25 bp was utilized to find centers, and selected windows with at least 50 fragment centers were designated a center cluster (CC). Utilizing these search parameters 18 875 CCs were discovered and 18,335 contained a consensus motif for CTCF when analyzed with the MEME motif discovery tool (Figure 4C) (32). For crosslinked CTCF DFF-ChIP, fragments of 50–100 bp in size were chosen, a scanning window of 100 bp was utilized, and selected windows with at least 50 fragment centers were chosen (Figure 4C). This analysis resulted in 32,905 CCs and 32 216 contained a consensus CTCF motif. These discovered motifs are both in strong agreement with the CTCF motif available from the JASPAR online database (Figure 4C).

To visualize the global occupancy of CTCF in DFF-ChIP and CUT&RUN, fragMaps were generated on the identified 18 335 native DFF-ChIP CTCF sites for each dataset centering on the 15 bp CTCF motif found (Figure 4D). The native DFF-ChIP fragMap shows a clear footprint of protection ranging from about 50–75 bp that is centered slightly upstream relative to the center of the CTCF motif. This footprint is due to CTCF alone protecting the underlying DNA. The shortest CTCF protections intrude more on the left side of the footprint indicating that the left side of the CTCF footprint is less well protected. Very similar results are apparent in the crosslinked DFF-ChIP fragMap over the same CTCF sites, with fragment sizes being slightly different due to impaired DFF digestion and the directional preference of nucleosome association being more apparent. CUT&RUN data show the smallest footprint for CTCF alone (40–70 bp) with the same upstream shift of the center of CTCF protections relative to the motif seen in DFF-ChIP. Surprisingly, despite slight detection of CTCF interaction with the right nucleosome, CUT&RUN primarily recovers the left rather than the right nucleosome. Whether this nucleosome retains any interaction with CTCF is unclear as there is no immunoprecipitation enrichment of soluble fragments in CUT&RUN protocols, merely the recovery of total digestion products.

Native DFF-ChIP recovers CTCF at occupancy levels that vary over three orders of magnitude and heatmaps were generated to get a sense of that scale since fragMaps do not show the variation and difference present at each site. Utilizing the 18,335 CTCF sites discovered using the native DFF-ChIP dataset, heatmaps of CTCF sites were generated and ordered based on signal over the motif (Figure 4E). As shown, DFF-ChIP recovers strong signal directly over and centered upon CTCF sites and shows the interactions with the left and right nucleosomes in both the native and crosslinked datasets. In comparison, CUT&RUN recovers mostly the surrounding nucleosomes rather than CTCF at each of the 18,335 sites. However, we considered the possibility that utilizing sites discovered using DFF-ChIP may not adequately represent the CUT&RUN data. To analyze the CUT&RUN dataset more fairly, DFF-CSP cannot be utilized since the majority of fragments generated by CUT&RUN are not located directly over the CTCF site. Therefore, FIMO from the MEME suite and the CTCF DFF-CSP motif (Figure 4C) were utilized to discover 1,048,505 CTCF binding motifs across the human genome. To select the occupied sites from the FIMO output, we analyzed only sites that had over 200 CUT&RUN reads covering the CTCF motif yielding 19,183 sites. Sorting from highest to lowest occupancy on the chosen CTCF sites resulted in a distribution that again shows the primary signal on many CTCF sites is the left nucleosome, not connected to CTCF (Figure 4F). However, CTCF protection alone is detectable over many of the sites. Interestingly, like we see in DFF-ChIP, CUT&RUN shows that the right nucleosome is generally closer to the CTCF motif, but the connection is rarely preserved since the majority of released nucleosomes lack any shared footprint with CTCF. Therefore, DFF-ChIP for CTCF provides unique benefits over CUT&RUN. Utilizing the same FIMO method to discover CTCF sites of occupancy in native and crosslinked DFF-ChIP resulted in 16,055 and 27,441 sites (Supplementary Data File).

### DFF-ChIP for GR reveals direct DNA bindings sites and sites driven by protein:protein interactions

To further explore the potential of the DFF-ChIP method, it was carried out targeting GR, a transcription factor whose binding is far less stable and specific than CTCF. GR is a ligand-induced transcription factor receptor that resides in the cytoplasm and then translocates to the nucleus upon glucocorticoid binding to alter transcription (45). In lymphoid cells, glucocorticoid treatment induces cell death and has been the cornerstone for treatment of lymphoid cancers for decades (46). As such, a B-cell precursor acute lymphoblastic leukemia cell line (SUP-B15) treated with 1 μM dexamethasone, a synthetic, potent, and specific glucocorticoid was utilized to induce GR translocation to the nucleus and those cells were then subjected to DFF-ChIP. Following 1 h of dex treatment, SUP-B15 cells were either crosslinked or not before isolation of nuclei. Duplicates for GR DFF-ChIP under native and crosslinked conditions had good correlations between replicas, native (*r* = 0.96) and crosslinked (*r* = 0.81) (Supplementary Figure S2). Additionally, nine GR ChIP-seq experiments with crosslinking were performed on SUP-B15s cells targeting GR with and without dex treatment. These experiments generated highly similar datasets (Supplementary Figure S4A) and for the remaining analyses the datasets were combined to yield very deep coverage of GR sites. There were essentially no specific signals for GR unless the cells were treated with dex (Supplementary Figure S4A), so the combined nodex data was subtracted from the combined dex data to generate the GR ChIP-Seq track.

**Figure 5.**
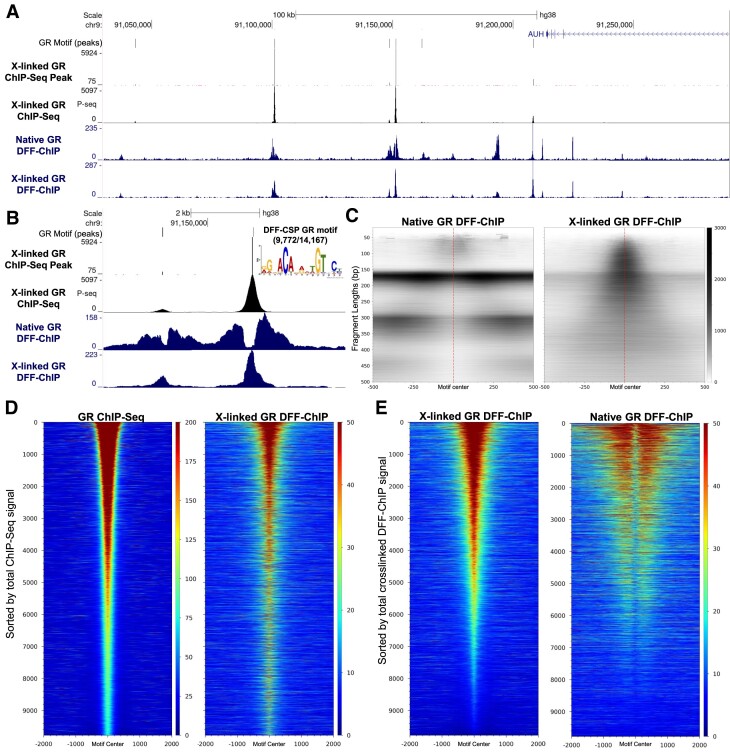
Analysis of GR DFF-ChIP over GR DNA binding sites. (**A**) UCSC genome browser track showing native and crosslinked GR DFF-ChIP, GR ChIP-Seq and the ChIP-Seq-Peak program called peaks (refer to Materials and Methods regarding input for peak calling). (**B**) Zoomed in view of the same tracks as in (A) focusing on two of the 9,772 peaks with a GR motif identified by the ChIP-Seq-Peak program. Additionally, the MEME motif found for 9,772 of the 14,167 identified peaks is shown. (**C**) FragMaps centered on the center of 9,772 GR motifs utilizing the native and crosslinked GR DFF-ChIP datasets. (**D**) Heatmaps centered on the 15 bp GR motif utilizing ChIP-Seq and crosslinked DFF-ChIP datasets sorted by coverage in the ChIP-Seq dataset ±500 bp at the 9,772 GR peaks with a GR motif. (**E**) Same as (D) except that the heatmaps utilized the crosslinked and native GR DFF-ChIP datasets sorted based on the crosslinked GR DFF-ChIP dataset ±500 bp of the 9,772 GR peaks with a GR motif.

Genome Browser tracks indicate that SUP-B15 GR ChIP-Seq gave strong peaks and both native and crosslinked DFF-ChIP show strong signal around these peaks (Figure 5A). However, native GR DFF-ChIP shows distinctive patterns of signal at these sites. To understand these sites on a global level, all peaks were found in the GR ChIP-Seq data using ChIP-Seq-Peak (47,48). This program utilizes ChIP-Seq data to accurately call peaks while retaining peak intensity; its use is described in detail in the Materials and Methods section. Peak calling and threshold cutoffs resulted in 14,187 peak calls and regions of 200 bp were selected around these peaks for MEME motif discovery which resulted in 9,772 peaks containing a consensus GR motif. Correlation analysis showed that all ChIP-Seq replicates correlated extremely well around these motif containing sites (Supplementary Figure S4B). A track showing the positions of occupied GR motifs and the GR ChIP-Seq Peak track are shown in Figure 5A. Closer examination of these sites reveals that under crosslinking conditions, both ChIP-Seq and DFF-ChIP show similar results with a peak centered upon a GR motif (Figure 5B). However, under native conditions, DFF-ChIP shows a reduced signal located precisely at the identified peaks and significant enrichment in the regions surrounding the site. Therefore, it seems that without crosslinking GR dissociates from its binding site, likely due to its low binding affinity (*K*_d_ = 10^−6^ to 5 × 10^−8^) (49) and the underlying sequence is digested by DFF. Based on these data, we suppose this transient association results in preferential enrichment of GR associated with the local chromatin leading to the inverse signal. FragMap representations of the 9,772 peaks with the GR motif also support this finding (Figure 5C). To determine how similar the crosslinked DFF-ChIP dataset is to the deep ChIP-Seq dataset, heatmaps for both were generated sorted by coverage over the 200 bp surrounding the 9,772 identified GR binding sites in the ChIP-Seq dataset. These heatmaps show good visual correlations (Figure 5D). Next, to analyze native DFF-ChIP data, heatmaps were sorted utilizing coverage in the crosslinked DFF-ChIP as the primary sort. These heatmaps again show that the native dataset has inverse signal immediately over the GR binding sites, but also that sites with the most GR occupancy have the most GR associated with the local chromatin (Figure 5E). Again, this supports the notion that after the binding site is vacated by GR and then destroyed by DFF, GR is left interacting with the local chromatin instead.

**Figure 6.**
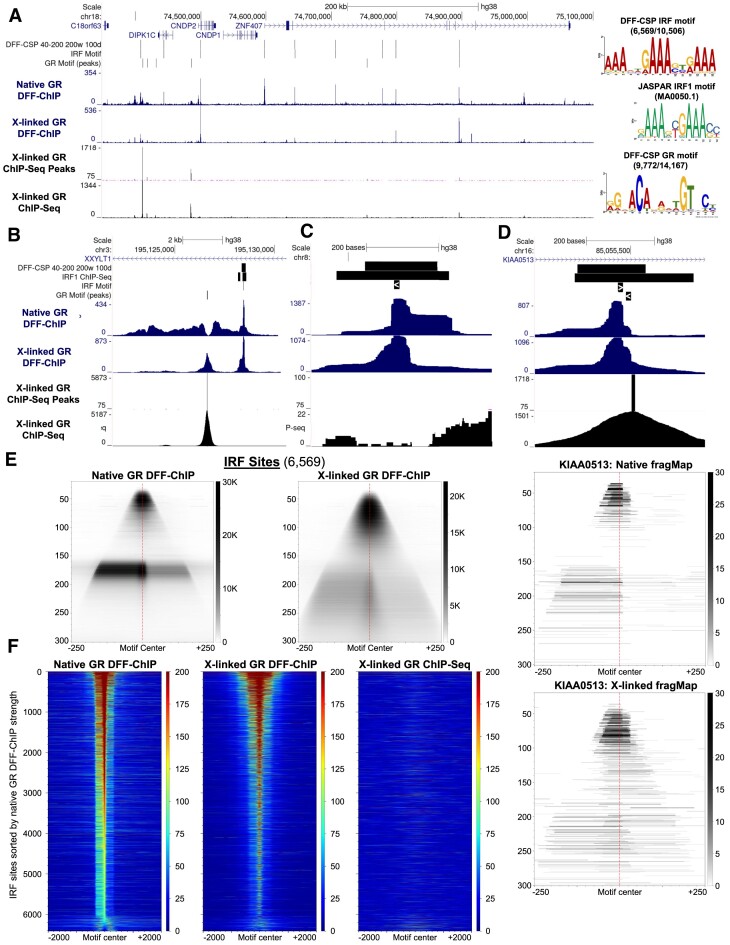
DFF-ChIP reveals GR tethered to IRF binding sites. (**A**) UCSC genome browser track showing GR DFF-ChIP, GR ChIP-Seq and the ChIP-Seq-Peak program called peaks. Sites unique to DFF-ChIP were identified utilizing DFF-CSP run on fragments of 40–200 bp in length from the native GR DFF-ChIP dataset with windows of 200 bp requiring at least 100 centers. The 10,506 resulting regions were used as input into the MEME motif discovery tool to identify 6569 center clusters containing the shown DFF-CSP IRF motif. Comparisons to the JASPAR IRF1 motif (MA0050.1) and the discovered DFF-CSP GR motif are shown. (**B**) Same as (A) highlighting a GR and IRF binding site. Also, regions enriched from IRF1 ChIP-Seq are also shown (85). (**C**) Same as (B) highlighting an IRF binding site with no detectable GR ChIP-Seq signal. (**D**) Same as (B) highlighting proximal IRF and GR binding sites. Below are native and crosslinked DFF-ChIP fragMaps depicting only this region highlighting two distinct footprints aligned with the marked IRF and GR motifs. (**E**) FragMaps centered on the 15 bp DFF-CSP IRF motif shown in (A) comparing native GR DFF-ChIP to crosslinked GR DFF-ChIP at the 6569 IRF Sites. (**F**) Heatmaps centered on the 6,569 15 bp DFF-CSP IRF motifs shown in (A) utilizing data from the native and crosslinked GR DFF-ChIP and the GR ChIP-Seq datasets. Heatmaps were sorted based on coverage of the 15 bp IRF motif in the native GR DFF-ChIP dataset.

GR DFF-ChIP also shows peaks under native and crosslinking conditions that are well replicated but do not exist in the ChIP-Seq data, such as those within the AUH gene (Figure 5A) and the many within the ZNF407 gene (Figure 6A). These peaks do not match the typical GR binding site signals as they are extremely well resolved, show no signs of clearing in the native dataset, and do not contain a GR motif. To make sense of these peaks unique to DFF-ChIP, we utilized the DFF-CSP program to identify regions enriched for fragment sizes common in many of these peaks. Initial visual inspection showed that these peaks commonly had a strong protection of about 40–60 bp in size but also had overlapping larger protections that indicate interaction with the local nucleosomes. Therefore, DFF-CSP was performed utilizing fragments sized 40–200, a scanning window of 200 bp, and a threshold of 100 fragment centers. Doing so resulted in 10,506 CCs and motif analysis returned a consensus motif of 15 bp composed of repeats of ‘GAAA’ separated by two bases derived from 6,569 of the input regions (Figure 6A). Comparison to known motifs utilizing TomTom from the MEME Suite showed that the most significant match was for interferon regulatory factor 1 (IRF1) which is capable of binding a single repeat of ‘GAAA’ (50). Additionally, IRF1 and other IRFs can work together to bind the discovered motif comprised of repeats of ‘GAAA’ and this is known as the interferon stimulated response element (ISRE). From the JASPAR database, the IRF1 motif shows strong correlation with our discovered motif and a clear distinction from the GR motif (Figure 6A, right).

The discovery of very strong IRF sites in DFF-ChIP targeting GR was surprising considering ChIP-Seq for GR has not uncovered such a finding. This again suggests a unique property of the DFF-ChIP method regarding the potential ability to preserve protein:protein interactions. Examples of IRF binding sites show the lack of signal over and surrounding IRF motifs in GR ChIP-Seq (Figure 6B, C). A track showing regions of IRF1 binding in breast cancer cells from IRF1 ChIP-Seq (51) is also shown in Figure 6B–D. In support of GR associating with IRFs, even though very different cell types were used and only one of the nine IRF proteins were targeted, 25% of the regions containing IRF motifs identified by GR DFF-ChIP were also identified by IRF1 ChIP-Seq. Standard ChIP-Seq utilizes harsh fragmentation via sonication and much more stringent washing with high salt and sodium deoxycholate containing buffers that would disrupt protein:protein interactions. This likely eliminated GR association with IRFs leading to loss of signal over IRF motifs while preserving GR crosslinked to the DNA. However, the GR ChIP-Seq utilized a different antibody (N499) than the one used for DFF-ChIP (IA-1). To determine if there was an issue with the antibody used, GR ChIP-Seq was carried out using the IA-1 antibody utilized in DFF-ChIP in replicate with very strong correlation between replicates (Supplementary Figure S4B). After visual interpretation and quantitative comparisons about direct GR binding sites and IRF sites, IRFs are only recovered in the DFF-ChIP method (Supplementary Figure S4C, D).

The ability of DFF-ChIP to resolve footprints of DNA bound proteins to such fine detail suggests that it may be possible to resolve simultaneous DNA binding of GR and IRF providing insights into their function. Such a feature is difficult to identify due to IRF binding commonly outweighing local GR binding profiles. However, an example of proximal IRF and GR binding sites shows that in ChIP-Seq a GR binding peak is clear, but that IRF dominates signal in DFF-ChIP (Figure 6D, top). FragMap analysis of this region utilizing native and crosslinked DFF-ChIP shows two distinct profiles of protection that align well with either the local IRF motif or the local GR motif (Figure 6D, bottom). This suggests two possibilities. First, IRF and GR are indeed co-bound to this region resulting in two distinct profiles. This possibility is likely, as crosslinking stabilizes the footprint overlapping the GR motif. A second possibility is that GR is still not bound and that IRF is showing two distinct bindings since the slight extension exists over a ‘GAAG’ sequence. Regardless, the resolution afforded by DFF-ChIP shows the potential to resolve overlap between bound transcription factors.

For global IRF analysis, fragMaps centered upon the 15 bp IRF motif were generated, and two major features were observed under both native and crosslinked conditions (Figure 6E). These represent protection from IRF proteins alone and their interaction with the neighboring nucleosomes. Native conditions give the most discrete features with IRF alone being 40–60 bp and the IRF-nucleosome being 160–180 bp. Crosslinking increased the average size of the features due to inhibition of DFF digestion. Like CTCF, IRF sites have a directional preference for association with a nucleosome. To determine if these fragMaps were representative of each IRF site, heatmaps were generated for each site and sorted based on coverage over the IRF motif in the native GR DFF-ChIP dataset (Figure 6F). Clearly, the preferential recovery of the left nucleosome is a feature of bound IRF and the majority of sites show this. Furthermore, standard ChIP-Seq completely fails to resolve any signal over these regions despite its considerably greater sequencing depth. Because detected IRF peaks associated with GR are largely unaffected by crosslinking, it is likely that these sites are recovered due to genuine GR tethering to IRF rather than redistribution of GR to IRF sites in the native condition.

**Figure 7.**
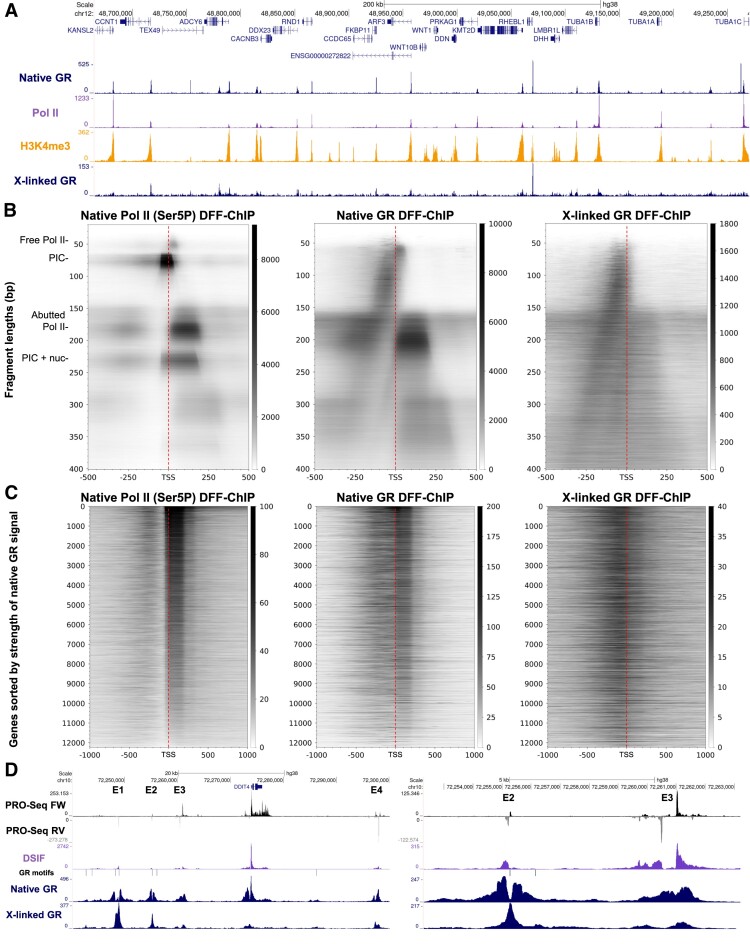
GR associates with engaged Pol II. (**A**) UCSC genome browser tracks showing DFF-ChIP targeting GR under both native and crosslinked conditions (SUP-B15 cells) with Pol II and H3K4me3 DFF-ChIP (HFFs). (**B**) FragMaps comparing DFF-ChIP targeting Pol II (Ser5P in HFFs) to native and crosslinked DFF-ChIP targeng GR (SUP-B15 cells). FragMaps are centered on the TSSs of 11,229 genes expressed in HFF cells discovered utilizing the truQuant program on a PRO-Cap dataset. (**C**) Heatmaps utilizing Pol II (Ser5P) DFF-ChIP and both crosslinked and native GR DFF-ChIP datasets sorted by abutted Pol II signal (+1 to +250) from the Ser5P dataset at the 12,229 genes expressed in HFF cells. (**D**) UCSC genome browser tracks showing four enhancers (E1–4) surrounding DDIT4. Tracks shown are PRO-Seq and DSIF (HeLa), native and crosslinked GR DFF-ChIP (SUP-B15 cells), as well as the GR ChIP-Seq peaks containing GR motifs.

Visual comparisons of native and crosslinked GR DFF-ChIP to previous DFF-ChIP experiments for transcribed regions targeting Pol II and H3K4me3 reveal many correlating peaks (Figure 7A). Crosslinked GR did not correlate as well as native GR with Pol II and H3K4me3 but was also typically enriched around promoters. To analyze promoter occupancy of GR in DFF-ChIP, fragMaps centered on the transcription start site of the 11,229 HFF truQuant genes were generated (Figure 7B). FragMap analysis of Pol II (Ser5P) DFF-ChIP shows PICs, PIC + nuc, and engaged Pol II that is either free or abutted to a nucleosome (Figure 7B). The free Pol II and abutted Pol II features are also found in the native GR DFF-ChIP fragMap, but not the PIC or PIC + nuc. The slightly larger size of the fragments in the abutted Pol II signal is likely due to the slight differences in DFF digestions of native and crosslinked nuclei. Native GR fragMaps also show enrichment of many different fragment sizes in the nucleosome depleted region (NDR) and this is the only feature significantly recovered in the crosslinked GR dataset. To ascertain GR levels at each gene, heatmaps were generated sorted by abutted Pol II signal in the Ser5P DFF-ChIP dataset (Figure 7C). Many of the analyzed promoters have NDR associated GR in both the native and crosslinked DFF-ChIP datasets and engaged Pol II signal specifically in the native DFF-ChIP dataset. This interaction with engaged Pol II could be direct or through associated elongation factors. The gene DDIT4 is surrounded by four prominent enhancers that function in a wide variety of cell types (Figure 7D, left). These enhancers display complex patterns of divergent transcription and also association of GR, but only some contain GR binding motifs. E2 contains a GR motif and gives the standard difference in occupancy between native and crosslinked DFF-ChIP signal (Figure 7D, right). E3 also has GR DFF-ChIP signal, but does not have a GR binding motif. The occupancy of GR under native conditions of E1-4 could be due to the interaction of GR with the engaged Pol II.

## Discussion

Our results show that DFF-ChIP provides an unprecedented level of precision compared to other methods of determining genome occupancy, and importantly, preserves protein:protein interactions crucial to transcription factor function. DFF-ChIP allows for precise positioning of promoter proximal paused Pol II and direct readout of the chromatin those complexes encounter (18). Here we interrogated how non-DNA bound factors responsible for modulating the activity of Pol II affect both. MED1 was globally associated with Pol II PICs and revealed initiated complexes retaining PIC contacts, whereas NELF and DSIF uncovered unique populations of paused Pol II complexes. Furthermore, the combination of performing DFF-ChIP under native and crosslinked conditions also provides possibilities for insights into the specific function of both strong and weak DNA binding transcription factors. CTCF DFF-ChIP revealed numerous occupied CTCF sites with slight directionality preferences for chromatin interactions, which is opposite to the nucleosome primarily recovered with CUT&RUN (20). GR DFF-ChIP uncovered protein:protein interactions that tether GR to chromatin flanking direct GR binding sites, IRF binding sites, and the transcription machinery near promoters, leading to insights impossible to achieve with other ChIP protocols.

The unique profiles uncovered in MED1 DFF-ChIP around promoter regions revealed insights into MED1 localization and function. Nearly every promoter analyzed had some level of MED1-associated PIC present indicating that this association is general, but the native DFF-ChIP failed to pick out regions bound by transcription factors that ought to interact with Mediator and subsequently be recovered via IP. These sites in promoter regions were revealed after crosslinking suggesting that the binding of the transcription factors to the DNA was not stable. Therefore, we favor the idea that Mediator is primarily brought to transcription complexes via interactions with the PIC. This line of thinking places the onus on transcription factors to find Mediator on PICs, not on Mediator to find them. Furthermore, our results suggest that at some promoters the loss of the upstream contacts of the PIC are not lost immediately upon initiation since we see features in both the MED1 data and the NELF and DSIF data with both the upstream PIC edge and downstream engaged Pol II. Such complexes have been observed in a yeast *in vitro* transcription system (52). Interestingly, these are more prevalent on TATA genes on which TFIID association is stabilized by an upstream TBP-TATA interaction. On Non-TATA genes, PIC extensions do not share a downstream edge with paused Pol II containing DSIF and NELF. During initiation PICs are locked into place and DNA is pulled in, leading to DNA unwinding and formation of the transcription bubble (53,54). The bubble itself would not be detected because DFF does not cleave single stranded regions, but scrunching of DNA into the PIC would lead to a downstream extension of DFF protection. Further investigations into this are necessary to establish how these features interplay with one another but an especially stable PIC scaffold may inhibit release of transcription complexes. Previous studies have also implicated GR to partially function through Mediator (55,56). We suspect this may contribute to the GR signal in the NDRs under crosslinked and native conditions.

The ability for DFF-ChIP to uncover directional nucleosome association of CTCF is significant as CTCF function is informed by the direction it binds to DNA with the strongest chromatin loops being formed by convergent CTCF sites (43,44). This preference arises due to CTCF’s N-terminus blocking cohesin-mediated loop extrusion significantly better than its C-terminus (57). Given that CTCF binds DNA with the N-terminus oriented towards the right-hand edge of the motif (58), the nucleosome detected with DFF-ChIP may be a contributing factor for the blockage of cohesin. Indeed, nucleosomes do inhibit the diffusion of cohesin (59). CUT&RUN recovers the opposite nucleosome as compared to DFF-ChIP and this is likely due to accessibility of cut-sites for the tethered MNase. The left nucleosome has a weaker interaction with CTCF according to DFF-ChIP and is clearly spaced further away from the CTCF site. This leaves both edges of the nucleosome available for cleavage. However, why the right nucleosome release is inhibited is unclear since its downstream edge should also be available for cutting. Regardless, because CUT&RUN only utilizes antibodies for targeted digestion rather than chromatin enrichment, fragments recovered are not necessarily associated with the target protein, rather they just happen to be nearby.

The data provided here concerning GR show that there are dramatic differences between ChIP-Seq, crosslinked DFF-ChIP, and native DFF-ChIP. ChIP-Seq detected GR only over GR DNA binding sites and this is likely due to the harsh conditions of the method that would eliminate protein:protein interactions. Similarly, crosslinked DFF-ChIP detected GR over the GR motifs, but with additional signals over IRF motifs and around promoters. Native DFF-ChIP also detected signal over IRF motifs and promoters, but in contrast, did not detect GR directly over GR binding motifs. Instead, native DFF-ChIP enriched for regions immediately surrounding GR motifs. GR is composed of a well-folded DNA-binding domain, a well-folded activation domain (the ligand binding domain/activation function 2) and a long N-terminal intrinsically disordered region (IDR) that is heavily post-translationally modified. With this structure, GR binds DNA with relatively modest affinity (*K*_d_ ≥ 10^−8^) (49). This likely leads to digestion of the GR binding motif during incubation with DFF leaving only protein:protein interactions for GR association with the chromatin.

The ability of DFF-ChIP to preserve protein interactions may provide the needed insights into the roles that GR plays to influence transcription. This work has examined GR binding in a B-ALL cell line, SUP-B15. Glucocorticoids are used for treatment of B-ALL by activating apoptotic pathways and repressing B-cell development pathways through regulation of transcription. We identified the consensus sequence GAAAnnGAAA beneath a large number of GR DFF-ChIP peaks that are not present in conventional GR ChIP-Seq. Because this motif is not directly bound by GR, we propose that GR is tethered to factors of the IRF family (60). Of the nine IRF family members (IRF1-9) six are expressed in SUP-B15 cells (IRF1,2,4,7,8,9) (61). Both IRF4 and IRF8 have been shown to play critical roles in B-cell development (62–64) and as suppressors of B-cell leukemia development (65,66). We find that of the IRF family members, IRF4 is critical for B-ALL survival and augments glucocorticoid-induced cell death (61,67). Among the IRFs, IRF4 has a consensus sequence (8) that matches the motif found under GR DFF-ChIP peaks well, suggesting a role of IRF4 in moderating the response of B-ALL to glucocorticoid-induced cell death. Thus, DFF-ChIP provides critical information to test the hypothesis that tethering of GR to IRF4 suppresses IRF4-activated genes critical for cell survival that augment the toxicity of glucocorticoids in B-ALL.

Native GR DFF-ChIP revealed interactions between GR and engaged Pol II around promoters. This association may be mediated be NELF which has been demonstrated to associate with NELF subunits (68–71). Previous studies have shown that recruitment of GR to heavily paused NFκB target genes influences the phosphorylation and occupancy of engaged Pol II primarily by preventing the function of P-TEFb (72,73). This suggests a mechanism of GR association with NELF on engaged Pol II to decrease P-TEFb function that may increase the fraction of NELF containing complexes. Such a function may be important for the effects that GR has at its binding sites. It has been shown that GR and other factors open chromatin to support bi-directional transcription about their binding sites (74). The resulting engaged Pol II which are under the influence of NELF can explain the association of GR with the regions surrounding occupied GR motifs. These findings highlight the importance of protein:protein interactions which is not completely surprising as prior work has already highlighted the importance of the GR IDR. These works show that when the IDR of GR is removed, GR loses ∼75% of its ChIP-Seq binding sites with the remaining binding sites diminished in occupancy (75). This could reflect the ability of the GR-IDR to tune the affinity of the GR-DBD (76), but a more likely explanation is that protein:protein interaction and direct readout of DNA work in concert to direct TFs to specific genomic loci. This is consistent with work from the Barkai lab showing that DBDs and IDRs work in concert to direct genomic localization of dozens of transcription factors in yeast (77–79). It has been recently proposed that IDRs promote association of TFs through nuclear condensates (80,81) that drive high local concentration of TFs near genomic loci. Additionally, it has been shown that GR has the potential to induce a redistribution of the local chromatin upon binding to DNA and the SWI/SNF chromatin remodeler is thought to partially drive this remodeling due to its associations with GR, its functional requirement for GR mediated transcription at many genes, and its co-occupancy following dex treatment (82–85).

Overall, DFF-ChIP is unmatched in its ability to uncover the exact context that DNA-binding factors encounter while associated with chromatin. We suspect that the preferential recovery of the many unique peaks in DFF-ChIP relative to ChIP-Seq has two primary causes. First, crosslinking between proteins can be less efficient than between DNA and proteins meaning that sonication is far more likely to disrupt these interactions. Secondly, native DFF-ChIP utilizes very mild conditions for ChIP and subsequent washing. Inclusion of high salt and detergents such as sodium deoxycholate may disrupt protein:protein interactions in traditional ChIP-Seq protocols, preventing detection of these important interactions. Further development of the method will likely lead to its application beyond the factors tested here and to optimization of its use for lower input samples. In addition, DFF-ChIP may be useful in dissecting the domains of transcription factors involved in specific protein:protein interactions after expression of these individual domains in cells. Despite the limitations we noted, CUT&RUN is still an impactful method that has advantages over DFF-ChIP for experiments with less precise aims and resources. CUT&RUN greatly limits the amount of sequencing required to capture the general regions of the genome associated with the target factor which greatly reduces sequencing costs. Furthermore, CUT&RUN returns interpretable results with as little as a few hundred cells as input making it a potentially more flexible protocol (19). As such, we expect DFF-ChIP and CUT&RUN to co-exist as two methods valuable to the progression of chromatin research.

## Supplementary Material

gkae760_Supplemental_Files

## Data Availability

Raw and processed PRO-Seq and DFF-ChIP data for this manuscript can be obtained from the GEO series GSE262859. Data we utilized generated by others can be obtained from the following GEO submissions: GSE84474, GSE163049, GSE155666, GSE156787. GreenCUT&RUN data were obtained from Sequence Read Archive SRP278136
