# Correction to ‘DFF-ChIP: a method to detect and quantify complex interactions between RNA polymerase II, transcription factors, and chromatin’

**DOI:** 10.1093/nar/gkag1272

**Published:** 2026-11-10

This is a correction to: Benjamin M Spector, Juan F Santana, Miles A Pufall, David H Price, DFF-ChIP: a method to detect and quantify complex interactions between RNA polymerase II, transcription factors, and chromatin, *Nucleic Acids Research*, Volume 52, Issue 18, 14 October 2024, Page e88, https://doi.org/10.1093/nar/gkae760

In the originally published paper, the x axis scale in panel D of Figure 4 was labeled incorrectly as −500 to + 500, the correct scale is −250 to + 250.

Figure 4 should read:

**Figure:**
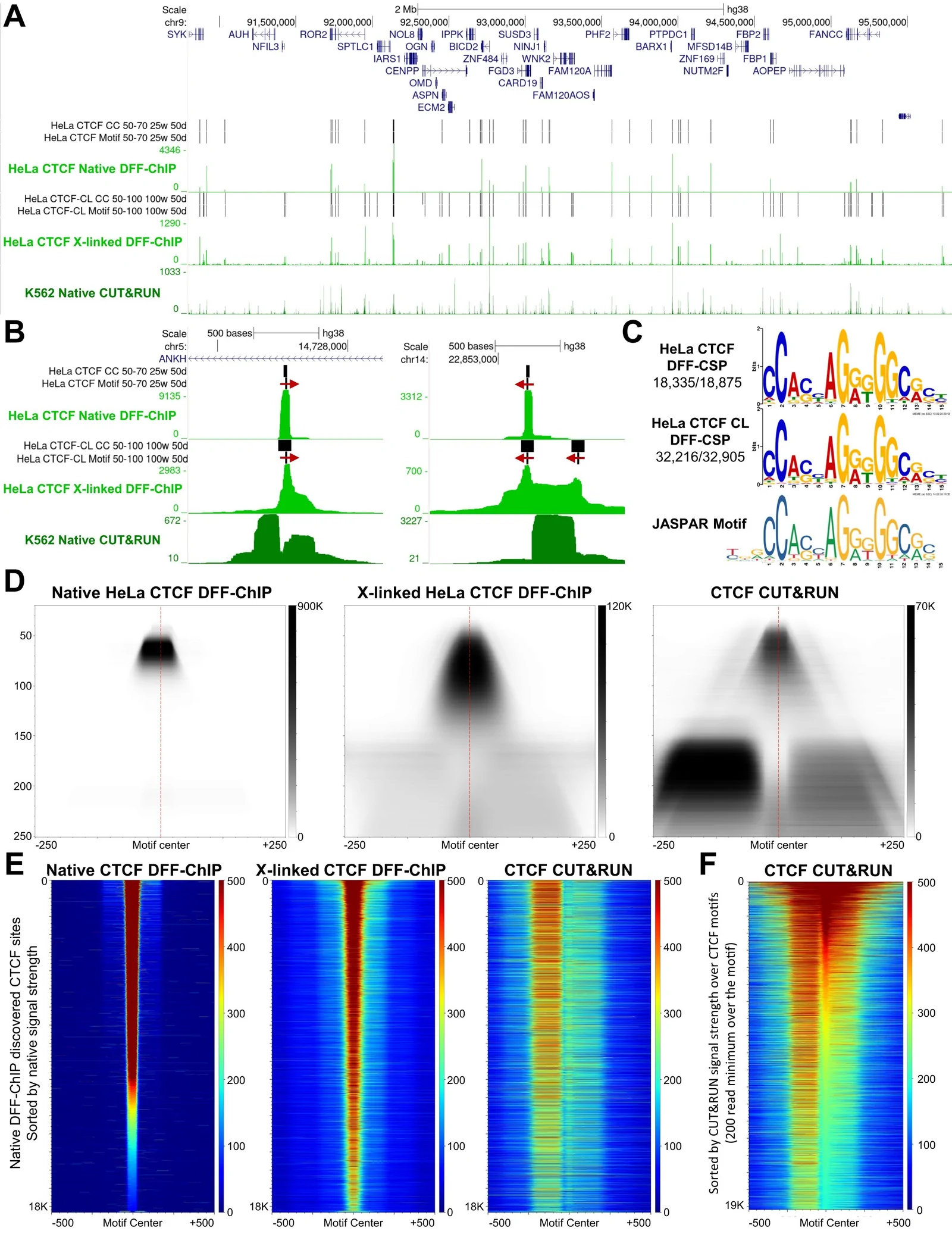


The error has been outlined only in this correction notice to preserve the version of record.

