# Supplementary material for: DFF-ChIP: a method to detect and quantify complex interactions between RNA polymerase II, transcription factors, and chromatin
Source: Nucleic Acids Res. 2024 Sep 9;52(18):e88. doi: 10.1093/nar/gkae760 (PMC11472042; doi:10.1093/nar/gkae760)
Supplement: gkae760_Supplemental_Files [file gkae760_supplemental_files.zip › Supplementary Figures.pdf]

DFF-ChIP: a method to detect and quantify complex interactions between RNA polymerase II, transcription factors, and chromatin

Benjamin M. Spector, Juan F. Santana, Miles A. Pufall, and David H. Price*

Department of Biochemistry and Molecular Biology, The University of Iowa, Iowa City, IA 52242, USA

Keywords: DFF-ChIP, CTCF, glucocorticoid receptor, DSIF, NELF, Mediator

*Corresponding author D.H.P. david-price@uiowa.edu

Supplementary Figures

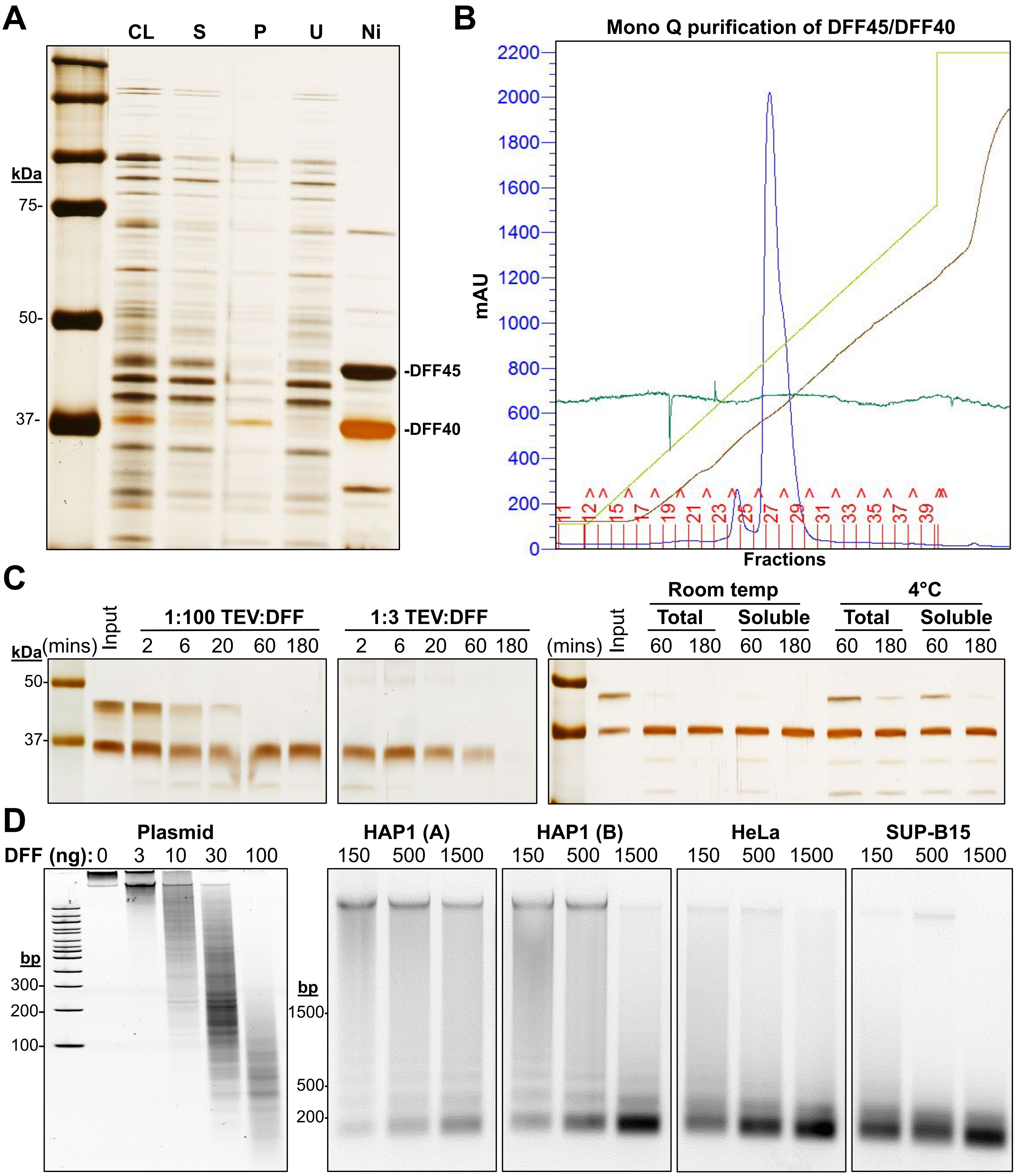
A
CL
S
P
U
Ni
kDa
75-
50-
37-
-DFF45
-DFF40
B
Mono Q purification of DFF45/DFF40
mAU
2200
2000
1800
1600
1400
1200
1000
800
600
400
200
0
11
12
15
17
19
21
23
25
27
29
31
33
35
37
39
Fractions
C
kDa
(mins)
Input
1:100 TEV:DFF
2 6 20 60 180
1:3 TEV:DFF
2 6 20 60 180
50-
37-
(mins)
Input
Room temp
Total
Soluble
60 180 60 180
4°C
Total
Soluble
60 180 60 180
D
Plasmid
DFF (ng): 0 3 10 30 100
bp
300-
200-
100-
HAP1 (A)
150 500 1500
HAP1 (B)
150 500 1500
HeLa
150 500 1500
SUP-B15
150 500 1500
bp
1500-
500-
200-

Supplementary Figure S1: Purification and testing of active DFF

(A) Silver stain showing steps during DFF40/45 heterodimer expression and purification. The gel shows the crude lysate (CL), supernatant (S) and pellet (P) after a 45 minute 45,000 rpm spin, the unbound material (U) after application to a nickel column, and the resulting nickel column elution (Ni). All, except for Ni, were loaded to equivalence. (B) Chromatogram showing the elution of the DFF40/45 heterodimer from the nickel elution loaded on Mono Q. DFF elutes at approximately 225 mM potassium chloride. The blue line indicates absorption units (mAU) and the brown line indicates conductivity. (C) Tests for optimal conditions for TEV digestion. The ratios of 1:3 and 1:100 of TEV protease to DFF heterodimer (weight/weight) were tested over a time course of digestion at room temperature (left gel). Room temperature and 4°C digestions were performed, and soluble material collected after centrifugation was compared to total material (right gel). (D) Test digestions of active DFF on plasmid DNA and nuclei from three different cell types. Each reaction contained 1 μg of a plasmid or 1 million nuclei with the indicated amounts of DFF. Digestions were for 30 minutes at 37°C.

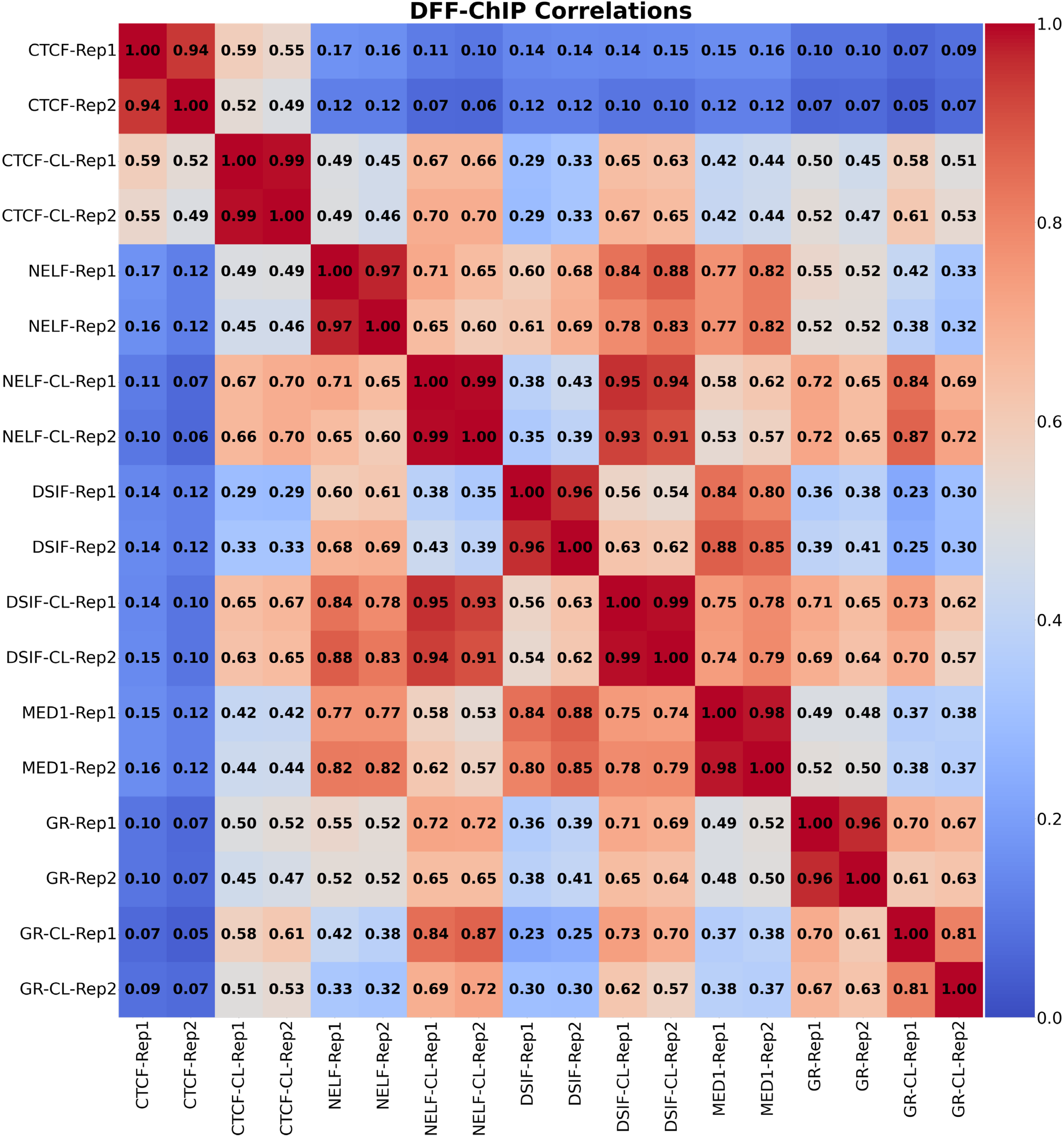
DFF-ChIP Correlations
CTCF-Rep1 1.00 0.94 0.59 0.55 0.17 0.16 0.11 0.10 0.14 0.14 0.14 0.15 0.15 0.16 0.10 0.10 0.07 0.09
CTCF-Rep2 0.94 1.00 0.52 0.49 0.12 0.12 0.07 0.06 0.12 0.12 0.10 0.10 0.12 0.12 0.07 0.07 0.05 0.07
CTCF-CL-Rep1 0.59 0.52 1.00 0.99 0.49 0.45 0.67 0.66 0.29 0.33 0.65 0.63 0.42 0.44 0.50 0.45 0.58 0.51
CTCF-CL-Rep2 0.55 0.49 0.99 1.00 0.49 0.46 0.70 0.70 0.29 0.33 0.67 0.65 0.42 0.44 0.52 0.47 0.61 0.53
NELF-Rep1 0.17 0.12 0.49 0.49 1.00 0.97 0.71 0.65 0.60 0.68 0.84 0.88 0.77 0.82 0.55 0.52 0.42 0.33
NELF-Rep2 0.16 0.12 0.45 0.46 0.97 1.00 0.65 0.60 0.61 0.69 0.78 0.83 0.77 0.82 0.52 0.52 0.38 0.32
NELF-CL-Rep1 0.11 0.07 0.67 0.70 0.71 0.65 1.00 0.99 0.38 0.43 0.95 0.94 0.58 0.62 0.72 0.65 0.84 0.69
NELF-CL-Rep2 0.10 0.06 0.66 0.70 0.65 0.60 0.99 1.00 0.35 0.39 0.93 0.91 0.53 0.57 0.72 0.65 0.87 0.72
DSIF-Rep1 0.14 0.12 0.29 0.29 0.60 0.61 0.38 0.35 1.00 0.96 0.56 0.54 0.84 0.80 0.36 0.38 0.23 0.30
DSIF-Rep2 0.14 0.12 0.33 0.33 0.68 0.69 0.43 0.39 0.96 1.00 0.63 0.62 0.88 0.85 0.39 0.41 0.25 0.30
DSIF-CL-Rep1 0.14 0.10 0.65 0.67 0.84 0.78 0.95 0.93 0.56 0.63 1.00 0.99 0.75 0.78 0.71 0.65 0.73 0.62
DSIF-CL-Rep2 0.15 0.10 0.63 0.65 0.88 0.83 0.94 0.91 0.54 0.62 0.99 1.00 0.74 0.79 0.69 0.64 0.70 0.57
MED1-Rep1 0.15 0.12 0.42 0.42 0.77 0.77 0.58 0.53 0.84 0.88 0.75 0.74 1.00 0.98 0.49 0.48 0.37 0.38
MED1-Rep2 0.16 0.12 0.44 0.44 0.82 0.82 0.62 0.57 0.80 0.85 0.78 0.79 0.98 1.00 0.52 0.50 0.38 0.37
GR-Rep1 0.10 0.07 0.50 0.52 0.55 0.52 0.72 0.72 0.36 0.39 0.71 0.69 0.49 0.52 1.00 0.96 0.70 0.67
GR-Rep2 0.10 0.07 0.45 0.47 0.52 0.52 0.65 0.65 0.38 0.41 0.65 0.64 0.48 0.50 0.96 1.00 0.61 0.63
GR-CL-Rep1 0.07 0.05 0.58 0.61 0.42 0.38 0.84 0.87 0.23 0.25 0.73 0.70 0.37 0.38 0.70 0.61 1.00 0.81
GR-CL-Rep2 0.09 0.07 0.51 0.53 0.33 0.32 0.69 0.72 0.30 0.30 0.62 0.57 0.38 0.37 0.67 0.63 0.81 1.00
CTCF-Rep1 CTCF-Rep2 CTCF-CL-Rep1 CTCF-CL-Rep2 NELF-Rep1 NELF-Rep2 NELF-CL-Rep1 NELF-CL-Rep2 DSIF-Rep1 DSIF-Rep2 DSIF-CL-Rep1 DSIF-CL-Rep2 MED1-Rep1 MED1-Rep2 GR-Rep1 GR-Rep2 GR-CL-Rep1 GR-CL-Rep2
1.0
0.8
0.6
0.4
0.2
0.0

Supplementary Figure S2: DFF-ChIP correlation analysis

Correlation analysis of all new DFF-ChIP datasets generated by dividing the genome into 10 kbp bins and summing reads from each bin. The Pearson correlations were then calculated for every dataset comparison possible.

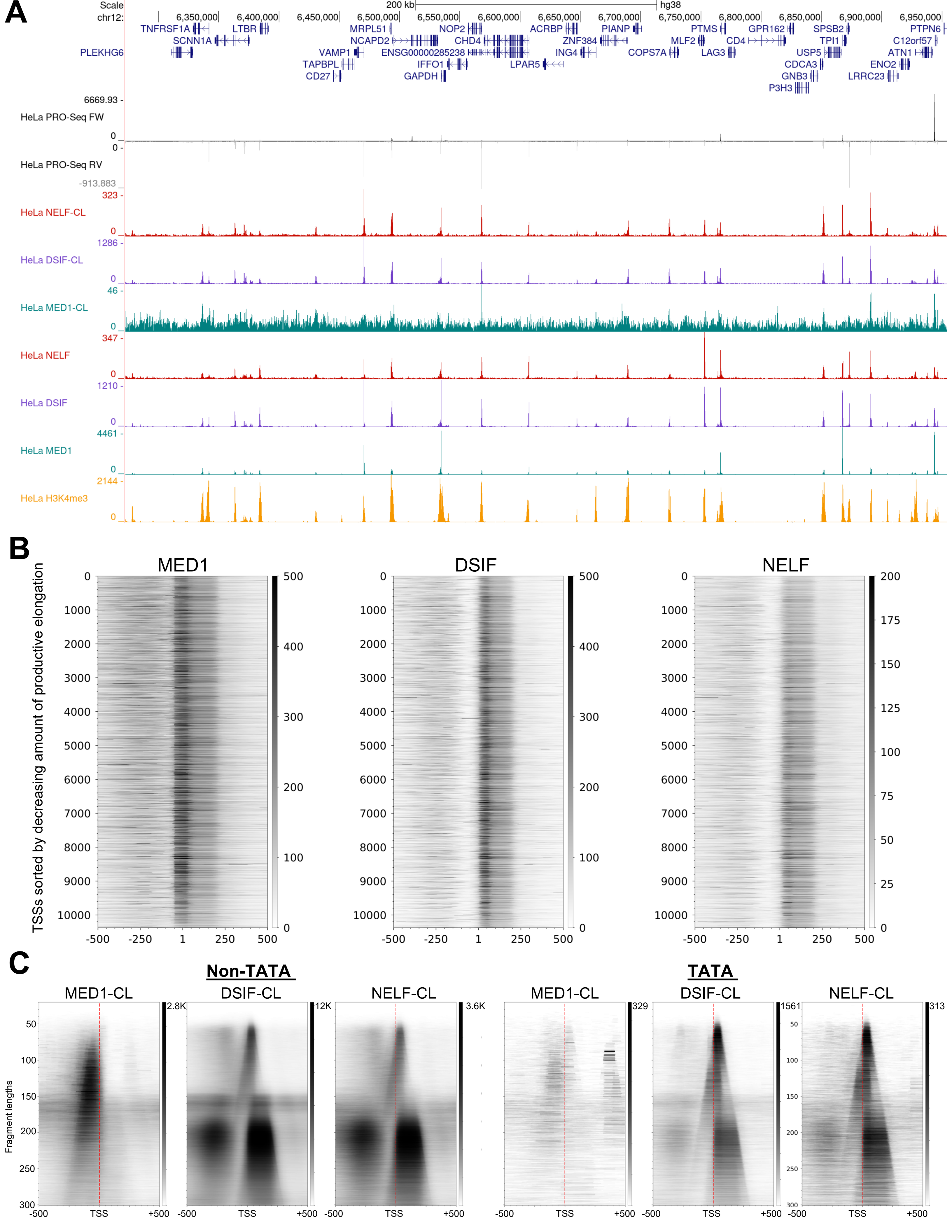
A
Scale
chr12:
200 kb
hg38
6,350,000
6,400,000
6,450,000
6,500,000
6,550,000
6,600,000
6,650,000
6,700,000
6,750,000
6,800,000
6,850,000
6,900,000
6,950,000
TNFRSF1A
LTBR
SCNN1A
PLEKHG6
MRPL51
NCAPD2
VAMP1
TAPBPL
CD27
NOP2
CHD4
ENSG00000285238
IFFO1
GAPDH
ACRBP
ZNF384
ING4
LPAR5
PIANP
COPS7A
PTMS
MLF2
LAG3
GPR162
CD4
SPSB2
TPI1
USP5
CDCA3
GNB3
P3H3
PTPN6
C12orf57
ATN1
ENO2
LRRC23
6669.93 -
HeLa PRO-Seq FW
HeLa PRO-Seq RV
-913.883
HeLa NELF-CL
HeLa DSIF-CL
HeLa MED1-CL
HeLa NELF
HeLa DSIF
HeLa MED1
HeLa H3K4me3
B
MED1
DSIF
NELF
TSSs sorted by decreasing amount of productive elongation
-500
-250
1
250
500
C
Non-TATA
TATA
MED1-CL
DSIF-CL
NELF-CL
Fragment lengths
2.8K
12K
3.6K
329
1561
313
TSS
+500

Supplementary Figure S3: Further analysis of DSIF, NELF, and MED1 DFF-ChIP

(A) UCSC genome browser tracks showing HeLa PRO-Seq and DFF-ChIP targeting DSIF, NELF, MED1, H3K4me3, and crosslinked MED1, DSIF and NELF. (B) Heatmaps of MED1, DSIF, and NELF DFF-ChIP signal around HeLa truQuant TSSs sorted by fraction of paused polymerases that enter productive elongation. 5’ ends of nascent transcripts found in the pause region (+/- 150 from weighted average TSS) were counted with and without flavopiridol treatment. The ratio of Flavo 5’ ends to control 5’ ends was calculated for each promoter region. A high ratio indicates a higher fraction of paused polymerases that enter productive elongation. (C) FragMaps for crosslinked MED1, DSIF, and NELF on Non-TATA and TATA promoters as in Figure 3.

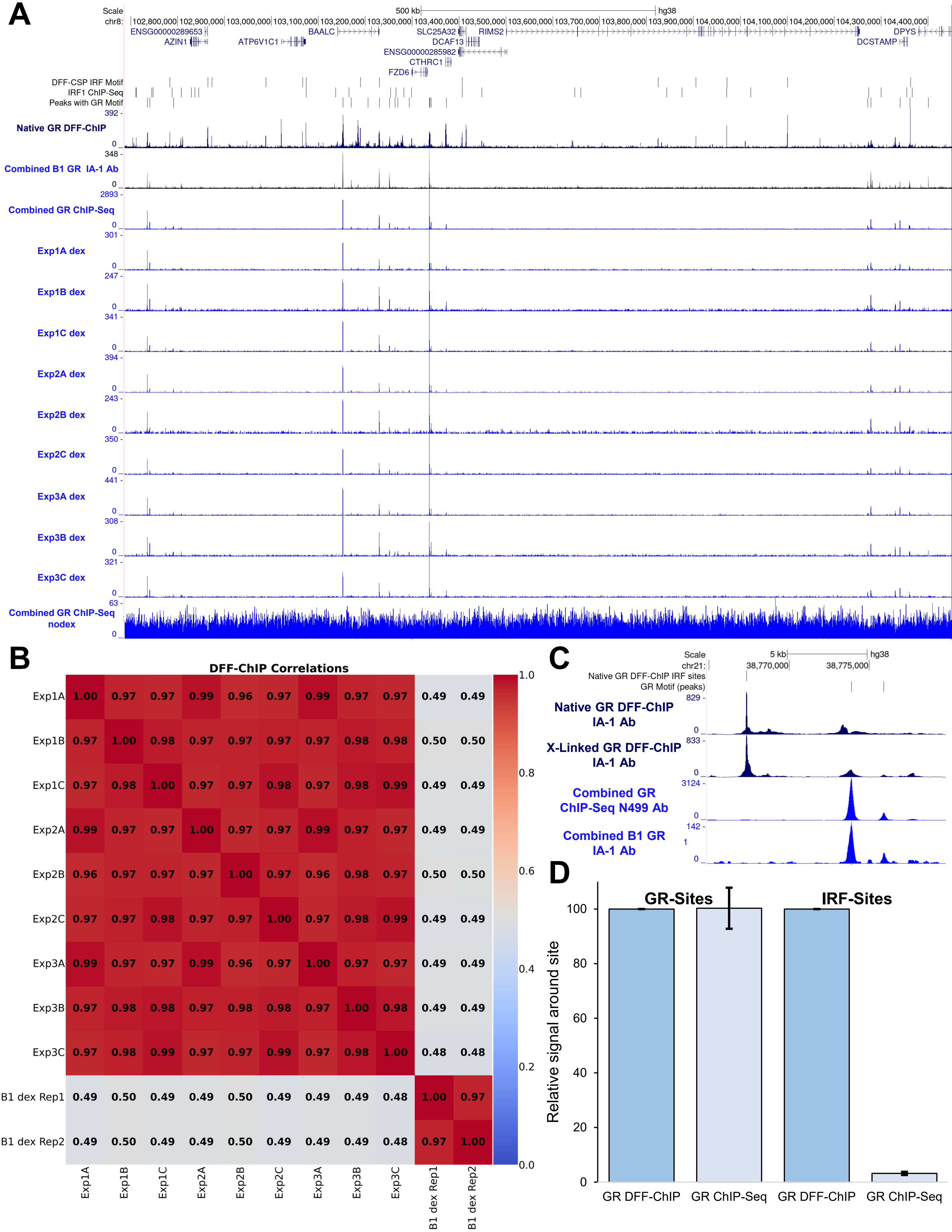
A
Scale
500 kb
hg38
chr8:
102,800,000 102,900,000 103,000,000 103,100,000 103,200,000 103,300,000 103,400,000 103,500,000 103,600,000 103,700,000 103,800,000 103,900,000 104,000,000 104,100,000 104,200,000 104,300,000 104,400,000
ENSG00000289653
AZIN1
ATP6V1C1
BAALC
SLC25A32
RIMS2
DCAF13
ENSG00000285982
CTHRC1
FZD6
DPYS
DCSTAMP
DFF-CSP IRF Motif
IRF1 ChIP-Seq
Peaks with GR Motif
392 -
Native GR DFF-ChIP
0
348 -
Combined B1 GR IA-1 Ab
0
2893 -
Combined GR ChIP-Seq
0
301 -
Exp1A dex
0
247 -
Exp1B dex
0
341 -
Exp1C dex
0
394 -
Exp2A dex
0
243 -
Exp2B dex
0
350 -
Exp2C dex
0
441 -
Exp3A dex
0
308 -
Exp3B dex
0
321 -
Exp3C dex
0
63 -
Combined GR ChIP-Seq nodex
0
B
DFF-ChIP Correlations
Exp1A 1.00 0.97 0.97 0.99 0.96 0.97 0.99 0.97 0.97 0.49 0.49
Exp1B 0.97 1.00 0.98 0.97 0.97 0.97 0.97 0.98 0.98 0.50 0.50
Exp1C 0.97 0.98 1.00 0.97 0.97 0.98 0.97 0.98 0.99 0.49 0.49
Exp2A 0.99 0.97 0.97 1.00 0.97 0.97 0.99 0.97 0.97 0.49 0.49
Exp2B 0.96 0.97 0.97 0.97 1.00 0.97 0.96 0.98 0.97 0.50 0.50
Exp2C 0.97 0.97 0.98 0.97 0.97 1.00 0.97 0.98 0.99 0.49 0.49
Exp3A 0.99 0.97 0.97 0.99 0.96 0.97 1.00 0.97 0.97 0.49 0.49
Exp3B 0.97 0.98 0.98 0.97 0.98 0.98 0.97 1.00 0.98 0.49 0.49
Exp3C 0.97 0.98 0.99 0.97 0.97 0.99 0.97 0.98 1.00 0.48 0.48
B1 dex Rep1 0.49 0.50 0.49 0.49 0.50 0.49 0.49 0.49 0.48 1.00 0.97
B1 dex Rep2 0.49 0.50 0.49 0.49 0.50 0.49 0.49 0.49 0.48 0.97 1.00
Exp1A Exp1B Exp1C Exp2A Exp2B Exp2C Exp3A Exp3B Exp3C B1 dex Rep1 B1 dex Rep2
1.0
0.8
0.6
0.4
0.2
0.0
C
Scale
5 kb
hg38
chr21:
38,770,000
38,775,000
Native GR DFF-ChIP IRF sites
GR Motif (peaks)
Native GR DFF-ChIP IA-1 Ab
829 -
0
X-Linked GR DFF-ChIP IA-1 Ab
833 -
0
Combined GR ChIP-Seq N499 Ab
3124 -
0
Combined B1 GR IA-1 Ab
142 -
1
0
D
GR-Sites
IRF-Sites
Relative signal around site
100
80
60
40
20
0
GR DFF-ChIP
GR ChIP-Seq
GR DFF-ChIP
GR ChIP-Seq

Supplementary Figure S4: GR ChIP-Seq analysis and IA-1 antibody validation

(A) UCSC genome browser tracks showing native GR DFF-ChIP compared to the total of all SUP-B15 ChIP-Seq +dex data after subtracting the no dex data, all individual replicates of GR ChIP-Seq with dex, and the combined GR ChIP-Seq with no dex. Additionally, the combination of two B1 GR dex ChIP-Seq replicates utilizing the same antibody utilized in DFF-ChIP (IA-1) is shown. (B) Correlations +/-100 bp around 9,773 GR sites for all GR ChIP-Seq datasets. (C) UCSC genome browser tracks highlighting the lack of signal around IRF sites in ChIP-Seq datasets with both the N499 and the IA-1 GR antibodies. (D) Comparison of signal recovery between IRF and GR sites from DFF-ChIP and ChIP-Seq utilizing the IA-1 antibody. The 9,773 GR sites were split into 10 groups and in each group DFF-ChIP coverage was set to 100% and the B1-dex data were multiplied by a total GR site signal normalization factor. The percentage of B1-dex relative to DFF-ChIP from each group was then found and used to find the average and standard deviation. This same method was applied to IRF site analysis except the normalization factor was not changed. More details are available in the Supplementary Data File.
